# Positive Impact of Increases in Condom Use among Female Sex Workers and Clients in a Medium HIV Prevalence Epidemic: Modelling Results from Project SIDA1/2/3 in Cotonou, Benin

**DOI:** 10.1371/journal.pone.0102643

**Published:** 2014-07-21

**Authors:** John R. Williams, Michel Alary, Catherine M. Lowndes, Luc Béhanzin, Annie-Claude Labbé, Séverin Anagonou, Marguerite Ndour, Isaac Minani, Clément Ahoussinou, Djimon Marcel Zannou, Marie-Claude Boily

**Affiliations:** 1 School of Public Health, Imperial College London, London, United Kingdom; 2 Unité de recherche en santé des populations, Centre hospitalier affilié universitaire de Québec, Département de médecine sociale et préventive, Faculté de médecine, Université Laval, Québec City, Québec, Canada; 3 London School of Hygiene & Tropical Medicine, London, United Kingdom; 4 Centre for Infectious Disease Surveillance and Control, Public Health England, London, United Kingdom; 5 Département de microbiologie, Hôpital Maisonneuve-Rosemont, Montréal, Québec, Canada; 6 Faculté des sciences de la santé, Université d'Abomey-Calavi, Cotonou, Bénin; 7 Centre national hospitalier universitaire, Cotonou, Bénin; 8 Population Services International, Cotonou, Bénin; 9 Dispensaire infections sexuellement transmissibles, Cotonou, Bénin; 10 Programme national de lutte contre le syndrome d'immunodéficience acquise et les infections sexuellement transmissibles, Cotonou, Bénin; McGill University Health Centre, McGill University, Canada

## Abstract

**Background:**

A comprehensive, HIV prevention programme (Projet Sida1/2/3) was implemented among female sex workers (FSWs) in Cotonou, Benin, in 1993 following which condom use among FSWs increased threefold between 1993 and 2008 while FSW HIV prevalence declined from 53.3% to 30.4%.

**Objective:**

Estimate the potential impact of the intervention on HIV prevalence/incidence in FSWs, clients and the general population in Cotonou, Benin.

**Methods and Findings:**

A transmission dynamics model parameterised with setting-specific bio-behavioural data was used within a Bayesian framework to fit the model and simulate HIV transmission in the high and low-risk population of Cotonou and to estimate HIV incidence and infections averted by SIDA1/2/3. Our model results suggest that prior to SIDA1/2/3 commercial sex had contributed directly or indirectly to 93% (84–98%) of all cumulative infections and that the observed decline in FSWs HIV prevalence was more consistent with the self-reported post-intervention increase in condom use by FSWs than a counterfactual assuming no change in condom use after 1993 (CF-1). Compared to the counterfactual (CF-1), the increase in condom use may have prevented 62% (52–71%) of new HIV infections among FSWs between 1993 and 2008 and 33% (20–46%) in the overall population.

**Conclusions:**

Our analysis provides plausible evidence that the post-intervention increase in condom use during commercial sex significantly reduced HIV prevalence and incidence among FSWs and general population. Sex worker interventions can be effective even in medium HIV prevalence epidemics and need to be sustained over the long-term.

## Introduction

As in other countries in West Africa, the HIV-1 epidemic in Benin has a high prevalence among female sex workers (FSWs) and, to a somewhat lesser extent, their clients. In the general population (GP) prevalence is lower than in countries in east and southern Africa [1] but is not, by any means, negligible. A comprehensive HIV intervention programme, Projet Sida 1/2/3 (SIDA1/2/3) funded by the Canadian International Development Agency, began in 1993 in the city of Cotonou, Benin. This included fully integrated field outreach activities focused on FSWs, which was extended to FSW clients in 2000 [2]. Cotonou is a rapidly expanding coastal city, with a population of approximately one million [3]. It is the largest city and the major economic centre of Benin. As a transport hub for commerce with the neighbouring countries of Togo, Nigeria and beyond, there is significant migration into Cotonou from and through these countries [4]. The majority of Cotonou's FSWs have been observed to be short- or long-term migrants from Togo and Nigeria and from Togo's western neighbour Ghana [3] (Fig S1).

The SIDA1/2/3 intervention programme focused on promoting consistent condom use among FSWs, promoting behavioural change, offering routine check-ups and free syndromic treatment of sexually transmitted infections [2]. The intervention was accompanied by a baseline round of interview-based bio-behavioural data collection at the start of the intervention in 1993 and five subsequent rounds over the next 15 years and four rounds among clients at FSW venues. In 1998 and 2008 bio-behavioural surveys were undertaken in the general population. The SIDA1/2/3 programme ended in 2006. Responsibility for the intervention was thereafter assumed by local health authorities [5]. Since then there has been anecdotal evidence suggesting the impetus behind the continuation of the intervention may have waned, resulting in condom use falling from peak levels.

**Figure 1:**
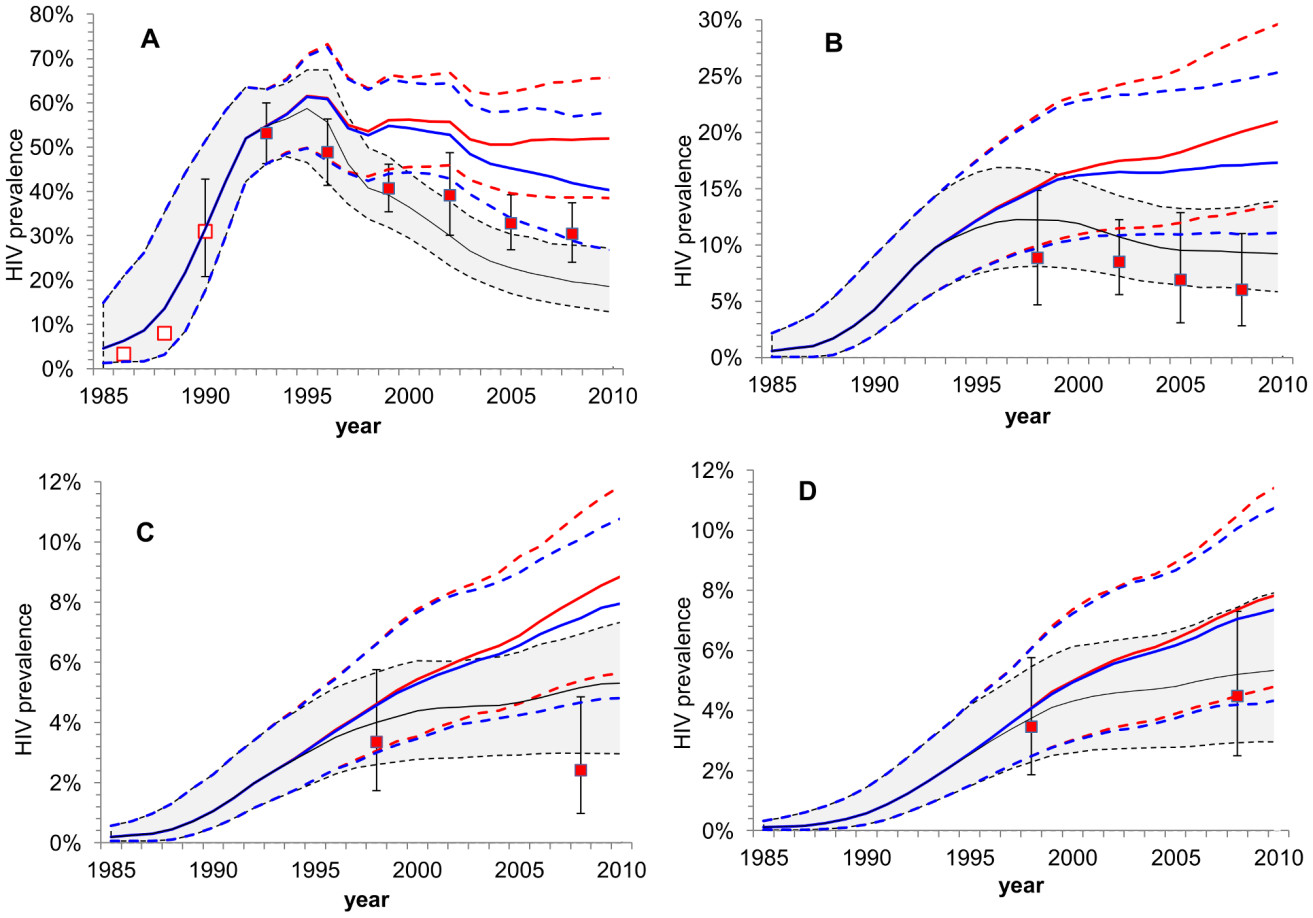
Observed and predicted HIV prevalence trends by risk groups. Observed and Predicted HIV prevalence among: A) all FSW nationalities combined, B) clients, C) all males (including FSW clients) and D) all females (excluding FSWs). Data points show the mean and 95% CIs of overall HIV prevalence from available data over time (solid red squares); open red squares (A) indicate data points for which CIs were not available. Solid lines (dashed lines) show the medians and 90%CrI (5th and 95th percentiles) of the modelling results. Good model fits under SIDA1/2/3 to the 38 targets used at the fitting stage (see methods) are shown as greyed areas. Also shown are results for the counterfactuals CF-1 (red), assuming FSW condom use remains at 1993 levels, and CF-2 (blue) assuming that FSW condom use mirrors that by moderate risk females in the general population. Note that overall HIV prevalence data, which were used to cross-validate model predictions because they were not used at the fitting stage (A, C & D), show good agreement with the modelled trends.

Following the start of the intervention in 1993 there was a threefold increase in condom use among FSWs, followed by substantial decline in HIV prevalence among FSWs in Cotonou [2], [6], from 53% in 1993 to 30% in 2008, and also in gonorrhoea prevalence. Additionally, declines in HIV prevalence from a representative sample of the general population were observed among FSW clients (2002–2008) and, between 1998 and 2008, among males, but not females (Fig 1) [7], [8].

The positive impact of large-scale interventions targeted to sex workers and their clients has been demonstrated in India, where HIV prevalence is low in the general population [9]. However, the impact of sex worker interventions in higher HIV prevalence settings remains somewhat controversial and/or under appreciated.

The main study objective was to assess the impact of the SIDA1/2/3 intervention on the heterosexual HIV epidemic in Cotonou, one which has been categorised as generalised according to the World Health Organisation classification of epidemics (because general population HIV prevalence exceeded 1% [10]). An additional objective was to estimate the contribution of commercial sex work to overall heterosexual HIV transmission. An HIV transmission dynamic model was parameterised and calibrated using multiple representative surveys of the general population, FSWs and clients. This was used within a Bayesian framework [9] to achieve two aims. Since self-reported data on condom use is susceptible to recall and social desirability bias, we first assessed the degree to which post-1993 HIV prevalence data support the recorded self-reported increase in condom use by FSWs in Cotonou during SIDA1/2/3 [6] (hypothesis testing), while taking into account the transmission dynamics of the population and the migration of FSWs, since both factors can influence HIV trends [11], [12]. The second was to estimate (taking into account parameter uncertainty) the impact on HIV prevalence, incidence and infections averted amongst FSWs and clients and the general population of Cotonou, of the self-reported increase in condom use by FSWs during commercial sex following the start of the SIDA-1/2/3 intervention. The strength of evidence provided by the hypothesis testing and modelling impact estimates are discussed and interpreted in the light of available information on implementation, coverage and intensity of the SIDA-1/2/3 and other interventions in Cotonou.

In addition to estimating the potential impact of the SIDA1/2/3 core group intervention on HIV in Cotonou, our mathematical modelling analysis adds to the evidence base on the effectiveness of interventions targeted to FSWs in higher prevalence settings than in classical concentrated epidemics such as in India where the effectiveness of these interventions have been shown [9], [13].

## Materials and Methods

### Mathematical Model

A 2-sex age-structured model of heterosexual HIV and gonorrhoea transmission [14] was developed (see flow diagram Fig S2 & equations Text S1). Demographic, behavioural and biological data representing the population of Cotonou, Benin were used to inform model parameter ranges and to fit the model to observed epidemiological trends as explained below (Table S1). The model stratified the male and female sexually active population into low and moderate behavioural risk groups, as well as four nationalities of FSW, and their male clients, each with age-specific rates of sexual contact (Table S1) and age-specific rates of sexual debut (Fig S3). The ‘low risk’ groups were defined as those who were married (or equivalent), with the remainder of the general population apart from clients classed as ‘moderate risk’. FSW risk groups represented Benin nationals and migrants from Ghana, Togo and Nigeria practising sex work. FSW migrants initially formed an estimated 98% of FSWs in Cotonou, declining to 80% during SIDA1/2/3(Fig S1) [2], [6]. The male clients were further divided in two groups representing “long-term” and “short-term” clients reflecting the fraction of clients who reported repeated contact with the same FSW over the past month (with or without contacts also with other FSWs) and those who did not; the numbers of acts between FSWs and short- and long-term clients were specified separately (Table S1). The model also allowed movement between all behavioural risk groups except for non-Beninese FSWs who were assumed to return to their country of origin after ceasing sex work in Cotonou (Fig S2). The model's representation of Cotonou demography was enhanced by incorporating age-specific fertility and background mortality rates appropriate to Benin which determined the age-distributions of individuals overall and by risk groups and the population growth rate (Fig S4 & Table S1).

The fraction of FSWs was assumed to remain constant over time (∼1.4% of all 15–49 females) [15]. The distribution of FSWs by nationality was assumed to remain stable between 1988 and 1993, in the absence of data to the contrary, and was allowed to change afterwards to reflect the observed and changing distribution of FSWs by nationality recorded during SIDA1/2/3, as this could influence HIV prevalence time trends even in absence of intervention and impact estimates. This was achieved by allowing the age-specific outgoing (estimated from estimates of duration of sex work from SIDA1/2/3 data) and incoming annual rates (Figure S5) of non-Beninese FSWs by nationality to vary over time (Table S1). Annual incoming rates were derived from the estimated outgoing rates, the number of FSWs by nationality and total fraction of FSWs. The HIV prevalence among non-Beninese FSWs at entry was allowed to vary over time at the parameter sampling stage to mimic changing overall HIV prevalence in their countries of origin (Figure S6); outgoing migration was assumed to occur independently of HIV status.

#### HIV

Newly HIV infected individuals were assumed to progress through an initially high infective primary infection stage followed by a long lasting ‘latent’ stage with reduced infectivity before progressing to a short pre-AIDS stage and finally to the AIDS stage (Fig S2). HIV infected individuals could leave the sexually active population at the same age-specific mortality rate as the rest of the population or due to AIDS specific mortality. The model allowed for specified proportions of individuals progressing to pre-AIDS (i.e. reaching CD4<350) or AIDS (CD4<200) to be treated and join an anti-retroviral treatment (ART) stage having low infectivity and enhanced survival. However treatment rates were calibrated only to the reported coverage of those eligible, defined as having CD4<200 in accordance with practice in the country over the period of the intervention [16], [17]. Treated HIV positive individuals could also fail or cease treatment, and return to the AIDS stage from which a proportion were allowed to re-initiate treatment.

#### Gonorrhoea

Gonorrhoea infection was modelled by assuming that individuals were either susceptible, infected and untreated, or infected and treated, and that those infected with gonorrhoea were at increased risk of HIV acquisition (Fig S2).

#### Force of infection

The force of infection (FOI), or annual rate of HIV (or gonorrhoea) infection per susceptible, was specific to each gender, age group (in the case of HIV) and activity class. It depended on: i) the infectivity per act of an infected contact within partnerships (allowed to vary by disease stage and, for HIV, gonorrhoea status); ii) fraction of sex acts protected by condoms at specified efficacy; iii) frequency of sex acts per sexual partner; iv) number and type of sexual partners; and v) mixing pattern (probability of selecting a partner from each specific risk group)(see below, Fig S7 and Text S1).

#### SIDA1/2/3 intervention and anti-retroviral treatment

**Figure 2:**
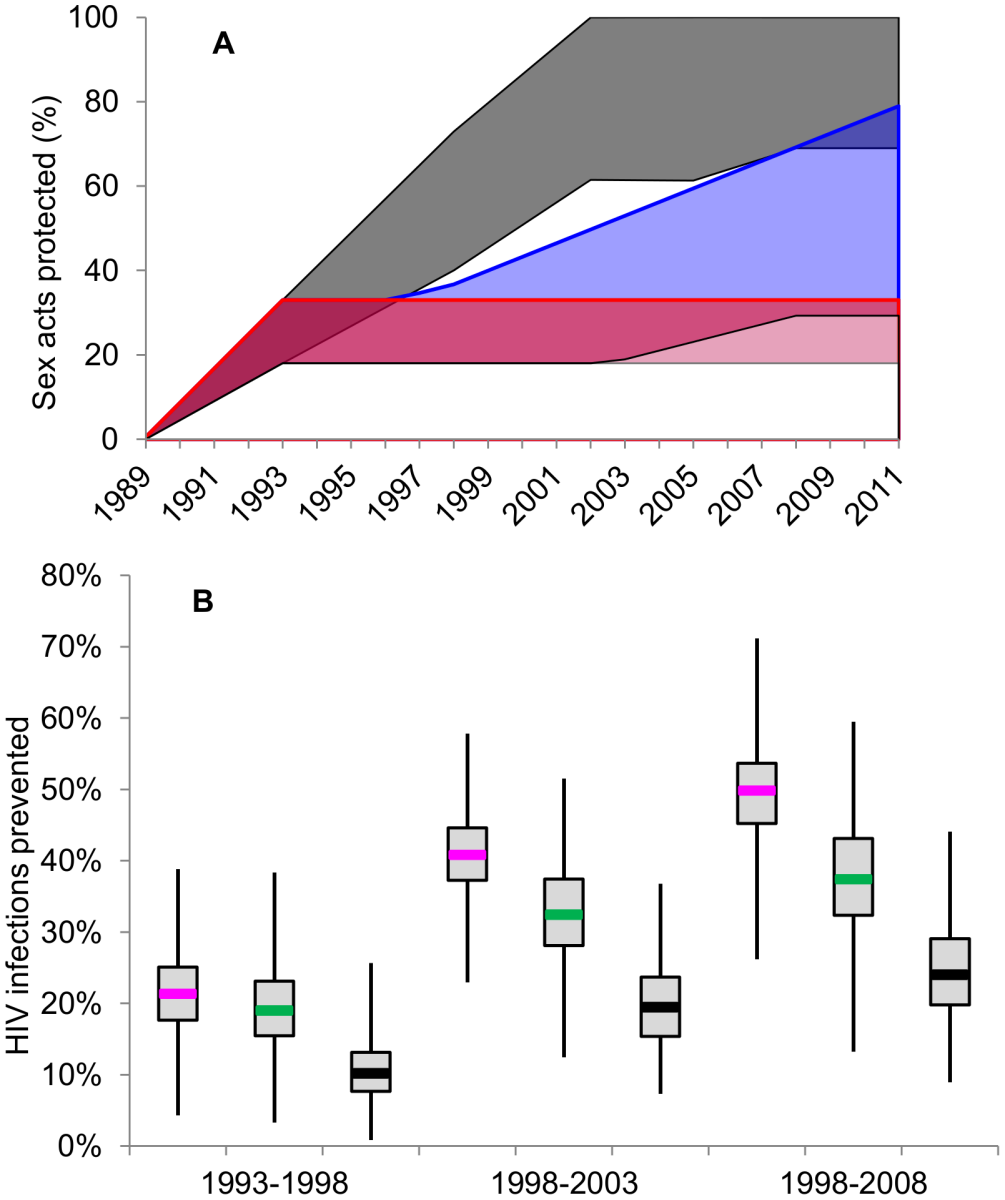
Condom use trends and infections prevented. A) 95%CrI of the modelled trends in condom use during: i) SIDA1/2/3 scenario (dark grey); ii) counterfactual CF-1 (red) in which FSW condom use remains at estimated 1993 level; and iii) counterfactual CF-2 (blue) in which FSW condom use mirrors that of the moderate risk females in the general population. Prior ranges assumed for condom use by FSW nationality (Table S1) reflect potential reporting biases in self reported condom use data. B) The proportion of HIV infections prevented under modelled SIDA1/2/3 condom use trends among FSWs (pink), clients (green) and the general population (black) compared to the counterfactual scenario (CF-2) cumulatively over 5 year time period: 1993-1998, 1998–2003, 2003–2008.

The fractions of acts between members of the different risk groups which were protected by condoms in the model was estimated as the proportions of males in the general population and FSW at different time points reporting use of a condom in the last sex act (Fig 2A). Condom use was assumed to increase linearly from zero in 1980 and then linearly between data points, to reflect time trends in reported condom use according to risk group following the start of the SIDA1/2/3 intervention in 1993; after 2008 condom use was assumed to plateau (Figure 2A) [2]. Uncertainty in the efficacy of condom use was taken into account by sampling over a pre-specified range of plausible values at the fitting stage as explained below (Table S1).

ART was initially introduced in Cotonou in 2002 [8], with WHO clinical stages III/IV or CD4 count ≤200 cells/mm3 determining eligibility during the whole SIDA1/2/3 period [18]. Data suggests that median CD4 cell count at ART initiation was similar for the general population at 115 (53–184) cells/mm3 compared with FSWs at 134 (67–175) cells/mm3 [18]. As data on ART uptake in Cotonou were quite sparse, we assumed that, from its introduction, uptake was the same for all risk groups in the general population and half of this for FSWs. Based on anecdotal evidence, we assumed also that uptake in the eligible general population in 2004 was below 10%. To reflect available data, ART uptake was parameterised as increasing linearly to 23–68% of HIV positives from the general population as they reach the eligibility criteria (WHO Guidelines for 2000:CD4<200 cells/mm3) in 2006 [16] and linearly again to 41–97% in 2009 (Table S1) [17]. Thereafter uptake remained constant at the 2009 level.

The effect of gonorrhoea treatment on duration of gonorrhoea infection, including treatment of FSWs and clients during SIDA1/2/3, was also incorporated in the model [19] (Table S1). We assumed that 55% of males and 25% of females were symptomatic of whom a fraction sought treatment. SIDA1/2/3 data on general population self-reported treatment of symptoms and on the periodic screening frequency of FSWs during SIDA1/2/3 were used to determine the fraction of gonorrhoea cases treated (Table S1). Under these assumptions the modelling results reflected the magnitude of decline in gonorrhoea among FSWs (results not shown).

#### Mixing patterns

For commercial sex, FSW and males who were clients were assumed to have contacts with each other in proportion to the number of contacts “offered” by each [20]. To reflect the pattern of contacts by age reported in the data (Figure S8), contacts between risk groups within the general population (i.e. excluding FSW but including males who happened also to be FSW clients) were defined as a mixing matrix partially assortative (“like with like”) by age. However additional assortativeness by risk group was imposed by constraints upon contacts within the general population matrix. Thus, low risk males (only reporting sex with spouse) could have only low risk female partners, whereas moderate risk males, and those who were also FSW clients, could have low and moderate risk female partners (Fig S9). Within these constraints a parameter determining the degree of assortativeness by age, following Garnett *et al*'s [20] method, was derived as part of the fitting process.

### Model calibration

First, we assigned ranges of plausible values to each of the model parameters using all site-specific data available, complemented with literature reviews (especially for the biological parameters) (Table S1). Only parameters determining initial demography, vital rates and the changing proportion of FSW of Benin origin over time during SIDA1/2/3 were specified as point estimates (Table S2 & Fig S1). The ranges of plausible setting-specific behavioural parameters were informed by the data collected during SIDA1/2/3 and representative surveys of the general population, which tested for HIV. Ranges for the biological parameters were based on relevant literature reviews and additional reports and official databases (e.g. US Census Bureau [21]). Second, we used Latin hypercube sampling (LHS) [22]–[24] to sample 500,000 combinations of parameters from the pre-specified data-driven ranges. The results of each run were compared with pre-specified HIV prevalence ranges derived from survey estimates. Parameter sets producing results agreeing with the targets (see following paragraph) were accepted as posterior parameter sets (these were not considered to be estimates for these parameters). We summarise results from the multiple runs by the 5th and 95th percentiles (referred to as the 90% Credibility Interval (90%CrI)).

The model was directly calibrated to 38 prevalence data points: 24 for FSWs (each of 4 nationalities for 1993, 1995, 1998, 2002, 2005, 2008), 4 data points for clients (1998, 2002, 2005, 2008) and 10 data points by gender and aggregate age groups for the general population in 1998 and 2008 (Table 1, Figures S9 & S10) [25]–[27]. Results were cross-validated to assess agreement with overall prevalence data not used at the fitting stage (Figure 1). Given the multiplicity of data sources, we accepted as good fits simulations that predicted HIV prevalence within target ranges wider than the 99% exact confidence intervals of the prevalence estimates by a fixed proportion of those intervals (see Text S2). Only runs that agreed with all the target constraints were accepted as a plausible parameter set.

**Table 1:** HIV prevalences (%) and number of seropositives (n) and total sample size (N) from SIDA1/2/3 data used in fitting the model: FSW, clients and general population males and females.

	HIV prevalence					
	(n/N)					
Year	1993	1995	1998	2002	2005	2008
**FSW Country of origin**						
Benin	57%	50%	20%	50%	33%	26%
	(4/7)	(16/32)	(26/131)	(2/4)	(25/76)	(19/72)
Ghana	56%	62%	58%	61%	51%	46%
	(135/240)	(74/120)	(74/127)	(33/54)	(32/63)	(17/37)
Togo	57%	68%	55%	65%	40%	36%
	(43/75)	(26/38)	(49/89)	(17/26)	(32/81)	(27/74)
Nigeria	27%	29%	37%	21%	23%	25%
	(11/41)	(33/115)	(83/223)	(22/105)	(40/173)	(35/139)
*Total*	*53%*	*50%*	*41%*	*39%*	*33%*	*30%*
	*(193/363)*	*(316/630)*	*(232/570)*	*(74/189)*	*(129/393)*	*(98/322)*
**CLIENTS**			8.5%	9.0%	6.9%	5.8%
			(34/401)	(54/601)	(20/288)	(18/310)
**GENERAL POPULATION**						
Males						
15–34			3.0%			0.5%
			(22/732)			(3/549)
35–49			4.6%			7.6%
			(9/194)			(15/197)
*Total males*			*3.3%*			*2.4%*
			*(31/926)*			*(18/746)*
**Females**						
15–19			2.4%			0%
			(5/212)			(0/148)
20–34			4.1%			5.2%
			(22/542)			(25/478)
35–49			3.1%			5.8%
			(12/383)			(13/223)
*Total females*			*3.4%*			*4.5%*
			*(39/1137)*			*(38/849)*

NB Cluster sampling was used for FSW, based on the most recent FSW mapping and enumeration, with sex work sites being sampled with a probability proportional to size FSW[6]; clients were sampled at FSW venues using a similar cluster sampling methodology [25]; the studies in the general population involved two-stage cluster sampling of a representative sample of households [8], [26], [27].

### Plan of analysis

We used the model to estimate the contribution of commercial sex work over time to overall HIV transmission between time *t_1_* and *t_2_* (i.e. the population attributable fraction, PAF(*t_1_*–*t_2_*)) defined as the relative difference using the multiple SIDA1/2/3 intervention parameter sets, between the predicted cumulative number of incident HIV infections, with and without FSW-client transmission over *t_1_* and *t_2_*. The annual fraction of infections due to commercial sex work is described similarly over individual periods of one year, PAF(*t_1_*–*t_1+1_*). Following an approach previously described [9], [19], we also used our Bayesian framework to assess if the decline in FSW HIV prevalence over time was more coherent with the trends in self-reported increase in condom use by FSWs during SIDA1/2/3 than when assuming no increases (CF-1) or a slower increases (CF-2) in condom use. Such an approach allowed us to assess the likely intervention impact independently of the natural transmission dynamics of infection (i.e. of AIDS mortality) or changes in FSW migration documented after the start of SIDA1/2/3 data)(Fig S1). This was done by repeating the fitting process, using the same prior ranges of parameter values of SIDA1/2/3, under two alternative counterfactual scenarios [9], [19], [28], assuming no, or slower, increases in condom use following the start of the intervention than currently suggested by data since 1993 (i.e. the SIDA1/2/3 trends). The first counterfactual (CF-1) assumed that condom use by FSWs followed the same assumption as for SIDA1/2/3 trends before 1993 (linearly increasing from a level in the range 0%–55% in 1989 to the 1993 SIDA1/2/3 level) but thereafter remained at the level reported in 1993 (prior value 18%–33%, Table S1) for all ensuing years (instead of reaching 60%–100% by 2002 (Fig 2A)). Counterfactual-2 (CF-2) assumed that condom use by FSWs remained at the level reported in 1993 until the time when use by moderate risk females reached the same level (the year varying depending on the sampled parameter); thereafter FSW condom use increased at the same rate as among moderate risk females until reaching a maximum of contacts protected within the range of 30%–70% in 2008 (Fig 2A). The relative frequencies of fits under the intervention and counterfactual scenarios were used to assess the plausibility of the hypothesis that the decline in HIV prevalence among FSWs was a result of the self-reported increase in condom use. Finally, the intervention impact was estimated by comparing the predicted cumulative number of infections between 1993 and 2008 (i.e. the course of SIDA1/2/3 program) under the SIDA1/2/3 condom use trends and under CF-1 and CF-2 in turn.

### Ethical considerations

We obtained entirely anonymous databases for this modelling study from the Benin National AIDS Control Program, the agency that owns these data. The overall impact assessment project, including the modelling as well as the 2008 data collection in FSWs, their clients and the general population, was approved by the ethics committee of the Centre hospitalier *affilié* universitaire de Québec. As there was no national ethics committee in Benin before 2008, for all surveys, ethical approval was obtained from *ad hoc* ethics committees convened by the Ministry of Health of Benin. In each survey, verbal informed consent was obtained separately for the interview and for the collection of biological specimens. Verbal consent was preferred to written consent to ensure full anonymity of the participants on all documents related to the study in the context of the stigma related to HIV and to the practice of sex work. The consent was documented on the consent forms by the signature of the interviewer.

## Results

### Model fits

Of the 500,000 parameter sets tested, 472 simultaneously agreed with all 38 targets (Figs S9-S10 & S12, Tables 2 & S1). Figure 1 shows that the predicted trends in overall HIV prevalence for the FSW, client and general populations reflected observed trends well even though, apart from clients, they were not fitted to these overall prevalence outcomes (fitting was to FSW prevalence by nationality and prevalence by aggregate age-bands for the general population). Figure 1 also shows the corresponding results for the two counterfactuals.

**Table 2:** Results of the hypothesis testing comparing number of fitting parameters to each condom trends hypothesis (SIDA1/2/3, CF-1 and CF-2)^1^.

*Fits to all data points up to and including this year*	*Number data points*	*Number of parameter sets producing good fits out of 500,000 parameter sets tested* 2		
		SIDA1/2/3	CF-1	CF-2
2008	10	472	0	0
2005	5	1,283	223	61
2002	5	2,270	2,198	522
1998	10	3,817	3,223	7,161
1995	4	17,593	14,268	14,696
1993	4	6,079	6,054	6,545
*Not fitting any data points*	0	468,486	474,034	471,015
*Total*		*500,000*	*500,000*	*500,000*

1 SIDA 1/2/3 = condom use reported by FSW; CF-1 = FSW condom use fixed at 1993 level in subsequent years; CF-2 = FSW condom use after 1993 mirrors that of moderate risk females.

2 Runs fitting the 38 data points, 1993 to 2008, for FSW, clients and the general population.

### Evidence for the self-reported increase in condom use

At the hypothesis testing stage, none of the different 500,000 parameter sets tested was able to simultaneously fit all FSW HIV prevalence data for all time points under the CF-1 or CF-2 trends (Table 2). The greatest discrimination between hypotheses occurred when there was information from more than 4 survey years (24 data points). This fitting suggests that the self-reported increase in condom use during SIDA1/2/3 was necessary to produce the decline in HIV prevalence among FSWs over time (472 fits to all data points obtained).

### Impact over the period of the intervention

**Figure 3:**
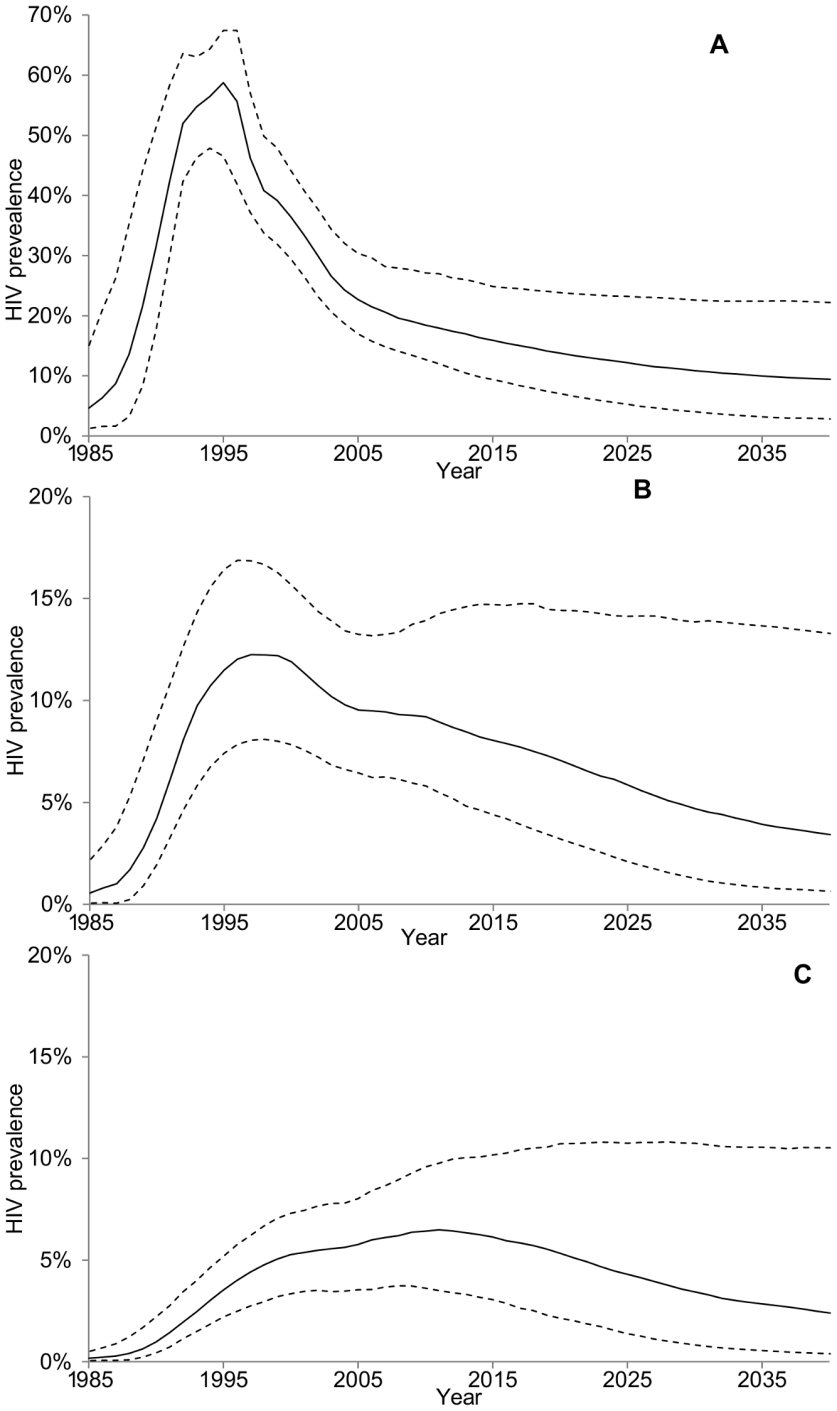
Long term impact on HIV prevalence of sustained condom use. Predicted HIV prevalence from the start of the epidemic to year 2040 among A) FSWs, B) clients and C) the general population assuming the pattern of condom use and migration patterns remains at current levels (median: solid line; 90%CrI: dashed lines).

The increase in condom use following the start of SIDA1/2/3 intervention and its impact on HIV prevalence compared to our conservative counterfactual CF-2 (assuming a smaller and later increase in condom use in absence of intervention), is shown in Figures 1 and 2A. The reported increase in condom use under SIDA1/2/3 may have prevented an estimated 50% (90%CrI 38–60%), 37% (90%CrI 25–50%) and 24% (90%CrI 14–37%) of new HIV infections between 1993–2008 among, respectively, FSWs, clients, and the total population excluding FSWs compared to CF-2(Figure 2B); and 63% (90%CrI 53–73%), 50% (90%CrI 35–62%) and 33% (90%CrI 20–46%) compared to CF-1(results not shown). Comparison of the predicted incidence rates of HIV infection among those susceptible (i.e. force of HIV infection) suggested that the increase in condom use during the intervention may have reduced the peak in the general population from approximately 1.4% person-year in 2002 (under CF-2) to 1.0%, a greater than 25% reduction from 2002 onwards (Figure S11). However, HIV incidence might have declined below 0.5% person-year if all HIV risk from commercial sex work could have been prevented from 1993 onwards. In addition, our model predicted that HIV incidence might have remained very low in absence of commercial sex work since the start of the HIV epidemic. This suggests that commercial sex is an important, if not the, main driver of the heterosexual epidemic in Cotonou despite HIV prevalence having reached more than 3% of the general population in the last decade. Therefore, in this context, it is clear that focusing prevention on sex workers is a necessary prevention strategy to control the HIV epidemic. If prevention efforts among FSWs were to be sustained at the same level, HIV incidence and prevalence could eventually reach these low levels within a few decades (Figures 3 & S11).

### Contribution of sex work to heterosexual HIV transmission in Cotonou

**Figure 4:**
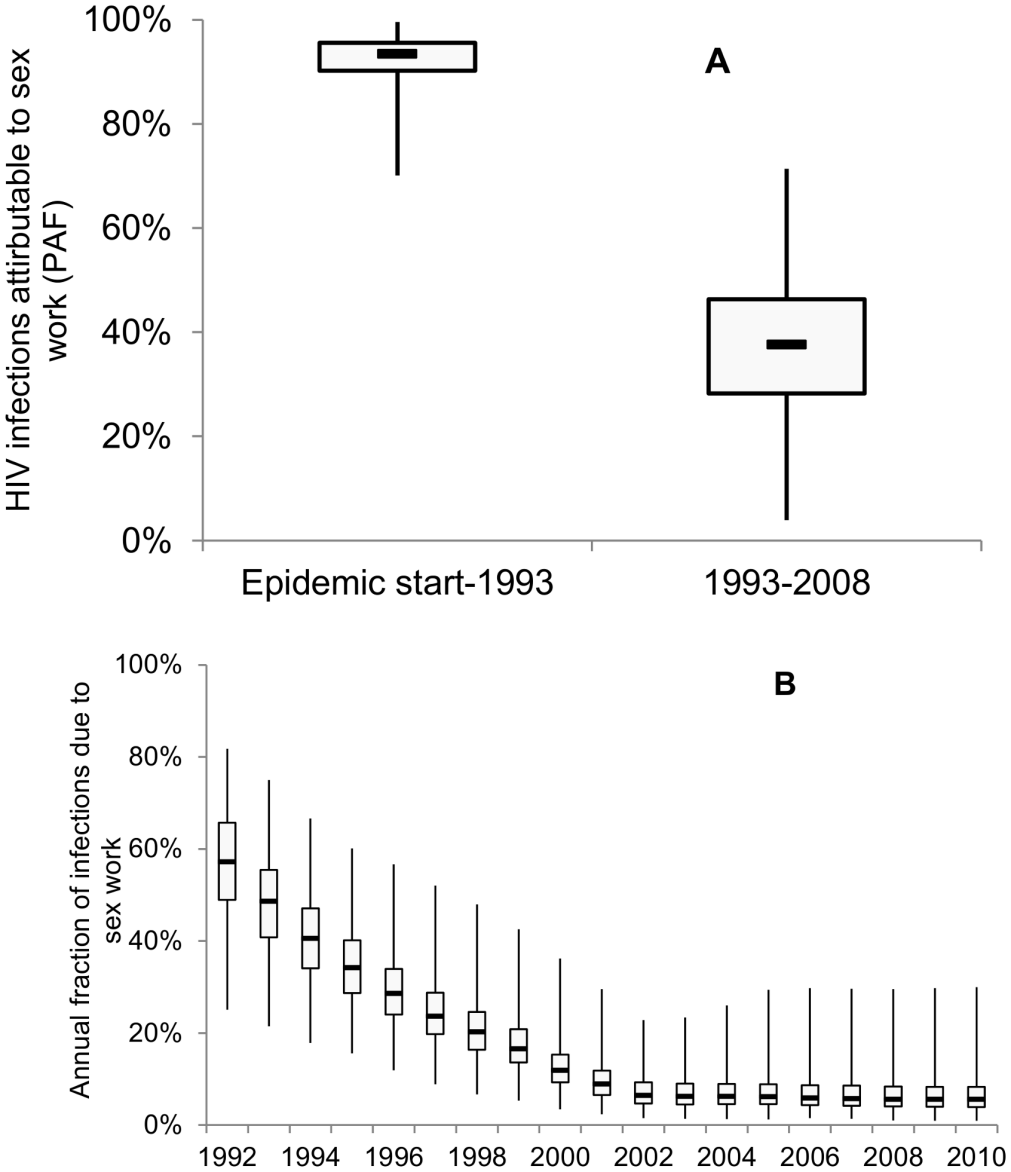
Contribution of commercial sex to heterosexual transmission. A) Cumulative population attributable fraction (PAF) calculated as described in Methods: i) from the start of the epidemic to the start of the intervention; and ii) from the start of the intervention in 1993 to 2008. B) Annual fraction of new HIV infections due to commercial sex work following the start of the SIDA1/2/3 intervention in 1993 (i.e. compares total number of new HIV infections in the general population every single year with and without FSW-client transmission). Box plots show the minimum and maximum, 25^th^ & 75^th^ percentiles and median.

Our results suggests that commercial sex may have contributed directly or indirectly to 93% (PAF(start-1993): 90%CrI 84–98%) of all cumulative heterosexual infections between the start of the epidemic and the start of the intervention and to 52% (PAF(1993–2008): 90%CrI 33–71%) of all infections between the start and the end of SIDA1/2/3 (Figure 4A). Figure 4B shows the annual fraction of HIV infections due to commercial sex work declined over time from a median of 57% in 1993 to 5–6% in 2010 as condom use increases and the epidemic matures (Figure 4B).

### Long-term impact

The results suggested that, if current rates of FSW condom use are maintained at 2008 levels until 2040, HIV prevalence would further reduce from 20% (90%CrI 14–27%) to 11% (90% CrI 3–23%) for FSW, from 10% (90%CrI 6–14%) to 4% (90%CrI 1–15%) for clients, and from 6% (4–9%) to 3% (0–11%) for the general population. It may also avert 72% (90%CrI 51–84%), 59% (90%CrI 30–78%) and 43% (90%CrI 16–66%) respectively of the new HIV infections between 2010–2040 compared to a new counterfactual that assumes that FSW condom use declines linearly from SIDA1/2/3 levels in 2010 to CF-1 level in 2015 and remains at this level thereafter (Fig 3).

## Discussion

In this study, we evaluated the SIDA1/2/3 intervention in Cotonou using mathematical modelling within a Bayesian framework to quantitatively assess the reliability of trends in condom used based on self-reported data from FSWs and to estimate the intervention impact independently of the underlying transmission dynamics of HIV and FSWs migration.

First, our modelling analysis has suggested that, since the beginning of the HIV epidemic until the start of the SIDA1/2/3 intervention, commercial sex work has contributed directly or indirectly (through long chains of onward HIV transmission) to most heterosexual HIV transmissions in Cotonou (median PAF(start-1993)  = 93%). However, the contribution of commercial sex work was lower during the fifteen years following the start of SIDA1/2/3 due to increases in condom use and the maturing of the HIV epidemic (median PAF(1993–2008)  = 52%). This highlights the importance of commercial sex even when HIV prevalence in the general population exceeds 1%, which was the WHO criterion typically used to classify a generalised HIV epidemic [10]. Our results support the argument that the heterosexual HIV epidemic in Cotonou is a concentrated one rather than a generalised epidemic with the main drivers being commercial sex work [11], [29], [30]. Our comparison of estimates of the annual and cumulative long-term PAF has also shown the difficulty in interpreting the short-term PAF (as typically reported in the literature [29]–[31]) as this underestimates the full contribution of sex work to overall transmission and the long-term prevention potential of targeted interventions. Our analysis has provided plausible evidence that the increases in condom use by FSWs reported during SIDA1/2/3 were sufficient and necessary to produce the observed decline in HIV prevalence; also that it may have averted a median 29% (18–40%) of all heterosexual HIV infections over 15 years of intervention.

Our study has several strengths. The modelling was based on extensive local data which were collected during SIDA 1/2/3 over a 15 year period and on general population surveys a decade apart. The model has been structured to reflect the main sources of the heterogeneity in the heterosexual population and incorporates age-specific and other processes varying through time as reflected by data. It also captures the influence of FSW cross-border migration in a setting where the majority of FSW are not local nationals. The combination of good data and dynamical modelling has allowed a better appreciation of the contribution of sex work to overall HIV transmission than when relying on classical methods to estimate PAF [33]–[35].

As with all modelling analysis and empirical data, our study also has some limitations. We did not include homosexual HIV transmission or transmission via intravenous drug use because data on these two risk population is extremely scarce in Benin [27], [36]. Therefore we may have slightly overestimated the contribution of sex work to overall heterosexual transmission. As the contribution of men who have sex with men (MSM) to heterosexual HIV epidemics in Africa has never been estimated, this is an important question to address in future. However, since many MSM may not reveal their identity, and may be married, some of them will have been included in our non-client general population surveys and their influence on HIV transmission among low to medium risk females may have been partly accounted for.

Self-reported data on sexual behaviour may be subject to recall or social desirability bias and could have influenced our estimates [27], [37]–[38]. However we accounted for this limitation in two ways. First prior parameter ranges for condom use were based on very wide confidence intervals for the data wider taking into account the possibility of some level of misreporting and/or faulty recall in either direction as suggested by a study comparing face-to-face interview and polling booth surveys, a more confidential interview method designed to reduce social desirability biases [27]. Thus our estimate of intervention impact takes into account some uncertainty in the level of condom use reported. Second, we conducted an analysis to test if the declines in FSW and client prevalence could have occurred in absence of the self-reported increase in condom use [13]. Uncertainty around parameters such as initial HIV prevalences among FSW, transmission probabilities, and degree of assortative mixing in the general population could have also biased our PAF estimates. However, our sensitivity analysis to understand the main drivers of the uncertainties suggested that only five parameters (that affecting year of HIV introduction, the initial prevalence of moderate risk males, and that of moderate risk females, the female *vs* male transmission ratio, and the weighting factor governing how any imbalances arising between general population male and female numbers of partners are reconciled, Table S3) were significantly, though relatively weakly, associated with the PAF estimates (Table S3). However as we also defined wide prior ranges for these parameters due to lack of data, our PAF estimates will reflect this uncertainty. The strength of our Bayesian approach is that it allowed taking into account uncertainty in parameter estimates since multiple parameters fits can be consistent with the data. However, since we varied and sampled many parameters at the fitting stage, this may have increased the uncertainty in our PAF and impact estimates.

As noted above, a major strength of our analysis is that it has been based on comprehensive epidemiological and behavioural data among FSWs and their clients collected over a period of fifteen years, which have been complemented by additional data from a large general population survey. These SIDA1/2/3 data suggested a marked decline in HIV and gonorrhoea prevalence among FSWs in Cotonou following the start of the intervention in 1993. Our Bayesian modelling analysis has suggested that the decline in HIV prevalence was not solely due to the natural transmission dynamics of infection or the rapid-turnover of the FSW population reported since 1993, but was predominantly due to the self-reported increases in condom use by FSWs following the intervention. While the rate of turnover in FSWs may have partly influenced the decline in HIV prevalence (when outgoing HIV infected FSWs may be replaced by incoming susceptible ones), the decline in FSW gonorrhoea prevalence over the same period (a much shorter duration infection less sensitive to migration but whose transmission is more sensitive to rapid increases in condom use [11], [32] supports the reported increase in condom use and subsequent impact on HIV transmission.

Although the model incorporated the potential influence of transnational FSW migration in and out of Cotonou, relevant data on key migration parameters such as HIV status of immigrant FSWs were scarce. Hence, migration parameter values were varied and selected at the fitting stage (Table S1) to reflect the distribution of FSWs by nationality. Sexual behaviour at each round of SIDA1/2/3 data collection and nationality-specific prevalence of incoming FSWs were also varied and selected during fitting. Thus this uncertainty was taken into account in model predictions of HIV trends. Initially, our results also relied heavily on the self-reported trends in condom use, which can be subject to recall and social desirability biases. However, our modelling hypotheses of different condom use trends, in the absence of a directly randomised or comparable control group, suggested that the self-reported condom use trends under SIDA1/2/3 were the most consistent with the HIV prevalence trends, strengthening their validity. Of the two counterfactuals, CF-1, which assumed that condom use would have plateaued at its baseline value in 1993 in absence of SIDA1/2/3, allowed us to estimate the total HIV infection averted due to the increase in condom use during SIDA1/2/3 without necessarily attributing all of the condom use increase to the prevention activities of SIDA1/2/3. Our hypothesis testing suggested that such a magnitude of increase was necessary to produce the FSW HIV prevalence decline since it was not possible to reproduce these trends under CF-1. CF-2 assumed that condom use between FSWs and clients would have followed similar increases as reported in the general population. However, due to the late and modest increases in condom use on which the CF-2 definition was based, the predicted impact of SIDA1/2/3 on HIV infections averted compared with CF-2 differed only modestly from that when comparing SIDA1/2/3 with CF-1.

Available data on the prevention programme suggest that the increase in condom use among FSWs is highly likely to have been due to the intervention under Projet SIDA-1/2/3 [5]. Indeed, from 1993 to 2006, it was the only intervention targeting FSW (and from 2002 clients) in Cotonou [6], [7]. Furthermore, until 2000, it was by far the largest externally supported intervention in the whole of Benin. This changed in the ensuing years, but without the implementation of any other intervention targeting FSW in Cotonou. Although the interventions initiated after 2000 [6] could have led to some increases in condom use in the general population, including among men who were clients of FSW, most of the increase in condom use in the sex work milieu could still be attributed to Projet Sida1/2/3 as most of the increase occurred between 1993 and the early 2000 s, as shown in Figure 2.

Despite some level of uncertainty regarding the long-term sexual behaviour and migration patterns of FSWs and the general population in Cotonou, our results also suggest that if the current levels of condom use are sustained, HIV prevalence and incidence could further decline among FSWs, and subsequently among clients and the general population. Although our modelling also included changes in ART availability in Cotonou, its impact would have been negligible during the period covered by SIDA1/2/3 since coverage was very low before 2004 and only increased toward the end of SIDA1/2/3. Thus, our study has highlighted the independent impact and continued importance of condom use interventions among FSWs and their clients.

Our results have important implications for our understanding of HIV epidemics in different contexts and support earlier model conclusions [11]. First, they suggested that commercial sex can be the main driver of HIV epidemics, meaning that without commercial sex HIV would have remained very low, even in epidemics which have reported relatively high HIV prevalence (>>>1%). This adds to previous analyses that have shown the impact of large-scale targeting of sex work in the context of populations as in India with low HIV prevalence in the general population (<1%) [9], [13]. Second, our results show that HIV targeted interventions are effective in reducing HIV prevalence and incidence among FSWs and their clients and, subsequently, in the general population even in higher prevalence settings. Similar results have been obtained for Kisumu, in Kenya [39]. However, when introduced in a mature epidemic, the impact of core group interventions occur in the longer term since the mechanism of action mostly relies on preventing short primary and longer secondary chains of transmission [11]. Therefore, FSW targeted intervention must be maintained in the longer term to be fully effective (i.e. until prevalence of HIV infectious individuals declines). Third, our results are also relevant to similar settings, especially in West Africa, with similar epidemic levels and sex work structure. In these settings, targeted commercial sex work interventions are a necessary component of HIV programmes to effectively and efficiently control the overall HIV epidemics. However, if more rapid impact is sought, intervention could also seek to target clients and then focus on the general population [11].

In summary, our study has provided additional evidence of the importance of scaling-up and sustaining current sex worker interventions in a wide range of epidemic contexts. The maximum impact of such interventions in the general population manifests itself in the longer term, especially when introduce in a mature epidemic. This has implications for the evaluation of intervention programmes, which should be evaluated on longer time scales than is typically the case (over 2–3 years). Finally, our results also highlight the contribution of mathematical modelling in the evaluation of interventions outside the context of randomised experiments.

## Supporting Information

Figure S1 **Relative proportions of main FSW nationalities.** Projet SIDA 1/2/3 data on relative proportions over time in Cotonou of FSWs from A) Benin, B) Ghana, C) Togo and D) Nigeria (red line & markers) compared with median (solid line), 5th and 95th percentiles (dashed lines) of model results. Note that numbers of Beninese FSWs in the model match the data exactly as their numbers were constrained within the model to match the relative proportion of Beninese FSWs among all FSWs recorded in the data.
(TIF)

Figure S2 **Flow charts of HIV/gonorrhoea model.** A) Flow chart describing the main features of the compartmental age-structured HIV (top) and gonorrhoea (gonorrhoea) (bottom) transmission dynamics models. Note that the gonorrhoea model is not age structured and runs in parallel with the HIV model in order to account for the co-factor effect of gonorrhoea infection on HIV transmission and model gonorrhoea trends; B) chart showing further elements of the model design: population flows between risk groups, sexual contact between risk groups and migration flows of female sex workers from neighbouring countries.
(TIF)

Figure S3 **Distributions of age- and gender-specific rates of sexual debut.** Examples of the range of triangularly distributed age-specific rates of sexual debut used in the model. Parameters of the triangular distribution (modal point, baseline value and scaling of the magnitude of the rates) were selected during LHS sampling of the model parameter set. Figure shows examples: A) with scale parameter taking values 0.1, 0.33 & 1.0 while modal point is kept constant at age group 25–29 and baseline value at 0.5; B) with modal point parameter taking values corresponding to 15–19, 25–29 & 30–34 age groups while scaling is kept constant at 0.33 and baseline value at 0.5; C) with baseline parameter taking values 0.005, 0.5, 0.9 & 1, 0.while modal point is kept constant at age group 25–29 and scaling at 0.33. In a similar way triangularly distributed age-dependent rates of change in risk group within the overall Beninese population, and corresponding parameters, were specified to and from each risk group (i.e. low and moderate risk males and clients, and low and moderate risk females and Benin FSW).
(TIF)

Figure S4 **Initial population age distributions.** Data (dotted line) and model projections (solid line) of population age distributions by 5 year age group for A) males and B) females for years 2002 (blue) and 2012 (red); and initial age distributions of each of the risk groups in the model: C) low risk (dotted line), moderate risk (solid line) and FSW clients (dashed line) males, D) low risk (dotted line) and moderate risk (dashed line) females in the general population and E) Beninese (solid line), Ghanaian (dotted line), Togolese (dashed line) and Nigerian (dot-dash line) FSWs.
(TIF)

Figure S5 **Age-specific distributions of FSW migration.** Examples of the range of triangular distributions used in the model to distribute migrating FSWs between 5 year age-groups. Parameters of the triangular distribution (modal point and shape, i.e. degree to which the triangular distribution is flattened) were selected during LHS sampling of the model parameter set with separate distributions used for Ghanaian, Togolese and Nigerian FSWs, and for inward and outward migration. Figure shows examples: A) modal point parameter taking values 15–19, 30–34 & 55–59 while shape parameter is kept constant at 0.5; B) with shape parameter taking values 0.0, 0.5 & 1.0 while modal point value is kept constant at the 30–34 age group.
(TIF)

Figure S6 **HIV prevalence of new entrant transnational FSWs.** Limits (solid black lines) of ranges of HIV prevalence of new entrant FSWs in the model: A) Ghanaian, B) Togolese and C) Nigerian. For each nationality, LHS sampling provided a linear rate of increase in prevalence and a subsequent rate of exponential decline following the year of change from linear increase to exponential decline (dotted black line). Corresponding 5 and 95 percentiles for the posterior fits are shown (solid red lines), and also minimum (blue diamonds) and maximum (red diamonds) prevalence data reported for the source countries [21]. Data points include general population as well as FSW data based on the assumption that new entrant FSWs may not begin SW until they have left their home region.
(TIF)

Figure S7 **Mixing structure.** Schematic diagram of pattern of contacts between risk groups which are allowed within the model. Note that males who are FSW clients may have non-commercial partnerships with other females whereas FSWs only have contacts with clients, some of whom may be “boy-friends”.
(TIF)

Figure S8 **Contact patterns from data.** Data illustrating the age distribution of partnerships with females in the general population of Cotonou reported by males who are also FSW clients (unpublished data from SIDA 1/2/3).
(TIF)

Figure S9 **Model HIV prevalence results compared with data targets.** HIV prevalence in different risk groups comparing model results and targets used in fitting the model: Results of each of the 472 model runs simultaneously fitting HIV prevalence predicted by the model to the 38 targets for the 4 FSW nationalities A) Benin, B) Ghana, C) Togo, D) Nigeria). Model results did not fit HIV prevalence for Beninese FSWs perfectly because the initially very low numbers of FSW reporting Beninese nationality resulted in very noisy trend estimates.
(TIF)

Figure S10 **Model HIV prevalence results compared with data targets.** HIV prevalence in different risk groups comparing model results and targets used in fitting the model: Results of each of the 472 model runs simultaneously fitting HIV prevalence predicted by the model to the 38 targets for A) clients, and general population females by age groups B) 15–19, C) 20–34, D)35–59, and males E) 15–34, F) 35–59.
(TIF)

Figure S11 **Force of HIV infection under different scenarios.** Time trends in the annual rate of HIV per susceptible person in the general population: i) based on Project SIDA 1/2/3 FSW condom trends assumptions (black); ii) under counterfactual scenario CF-2 (blue); iii) assuming absolutely no transmission during commercial sex from 1993 (i.e. akin to a perfect intervention that would have started in 1993) (pink); and finally, iv) assuming absolutely no transmission during commercial sex since the start of the HIV epidemic (dashed pink).
(TIF)

Figure S12 **Selected distributions of posterior parameter ranges.** Examples of the shapes of the distributions of the posterior parameter ranges shown in Table S1 (the associated skew values shown in the table are repeated in brackets after the title of each distribution).
(TIF)

Table S1 **Prior and posterior parameter ranges.** S1a: Biological parameters. S1b: Intervention and treatment parameters. S1c: Behavioural parameters. S1d: Initial prevalence parameters. S1f: Migration.
(DOC)

Table S2 **Demographic parameters.** S2a: Estimated net rates of change in FSW populations per year by nationality. S2b: Demography.
(DOC)

Table S3 **Correlations between PAFs and model parameters.**
(DOC)

Text S1 **Model equations.**
(DOC)

Text S2 **Further modelling details.**
(DOC)
