# Supplementary figures and images for: Positive Impact of Increases in Condom Use among Female Sex Workers and Clients in a Medium HIV Prevalence Epidemic: Modelling Results from Project SIDA1/2/3 in Cotonou, Benin
Source: PLoS One. 2014 Jul 21;9(7):e102643. doi: 10.1371/journal.pone.0102643 (PMC4105482; doi:10.1371/journal.pone.0102643)

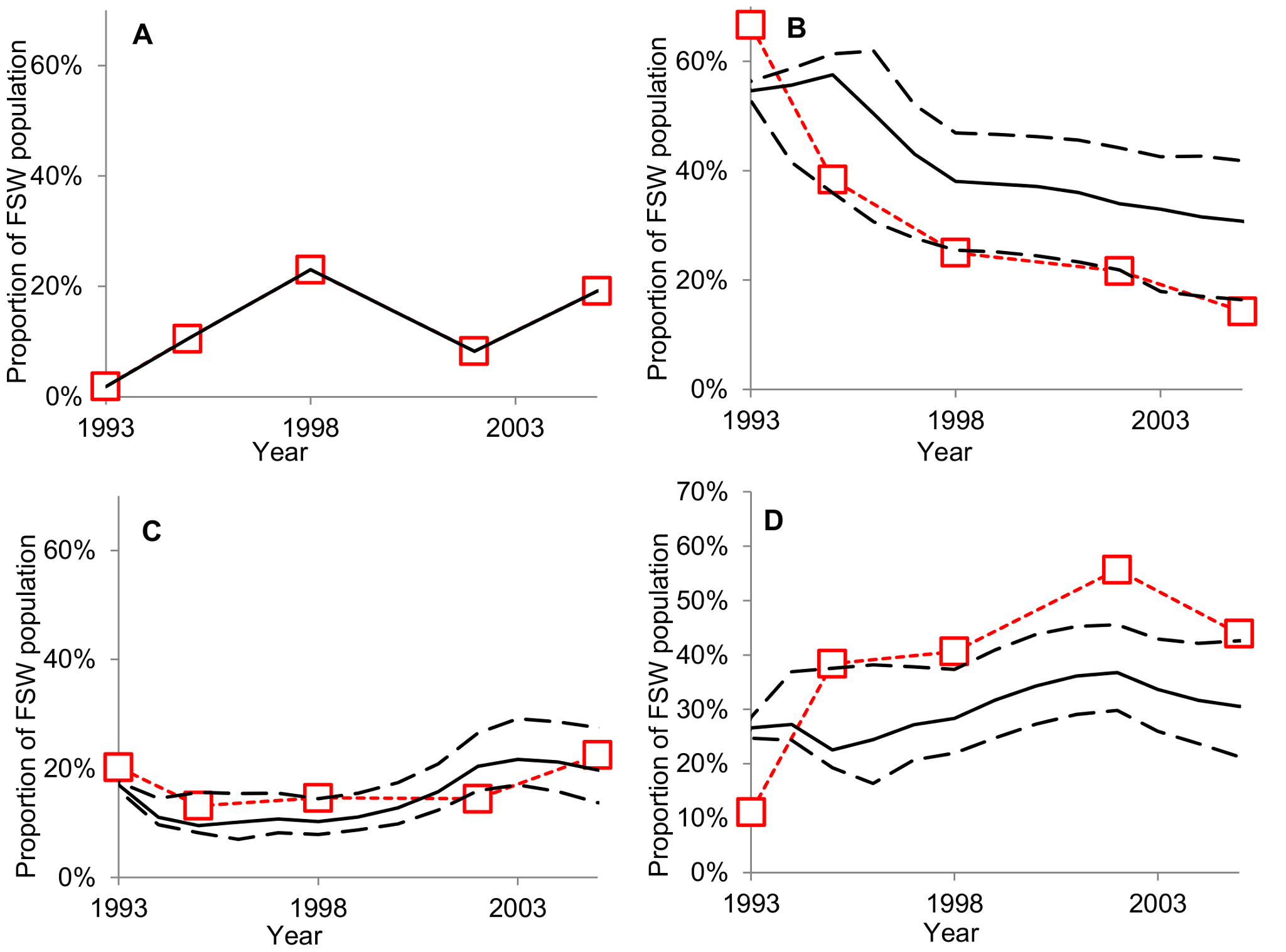

Supplement: Figure S1 — Relative proportions of main FSW nationalities. Projet SIDA 1/2/3 data on relative proportions over time in Cotonou of FSWs from A) Benin, B) Ghana, C) Togo and D) Nigeria (red line & markers) compared with median (solid line), 5th and 95th percentiles (dashed lines) of model results. Note that numbers of Beninese FSWs in the model match the data exactly as their numbers were constrained within the model to match the relative proportion of Beninese FSWs among all FSWs recorded in the data. (TIF) [file pone.0102643.s001.tif]

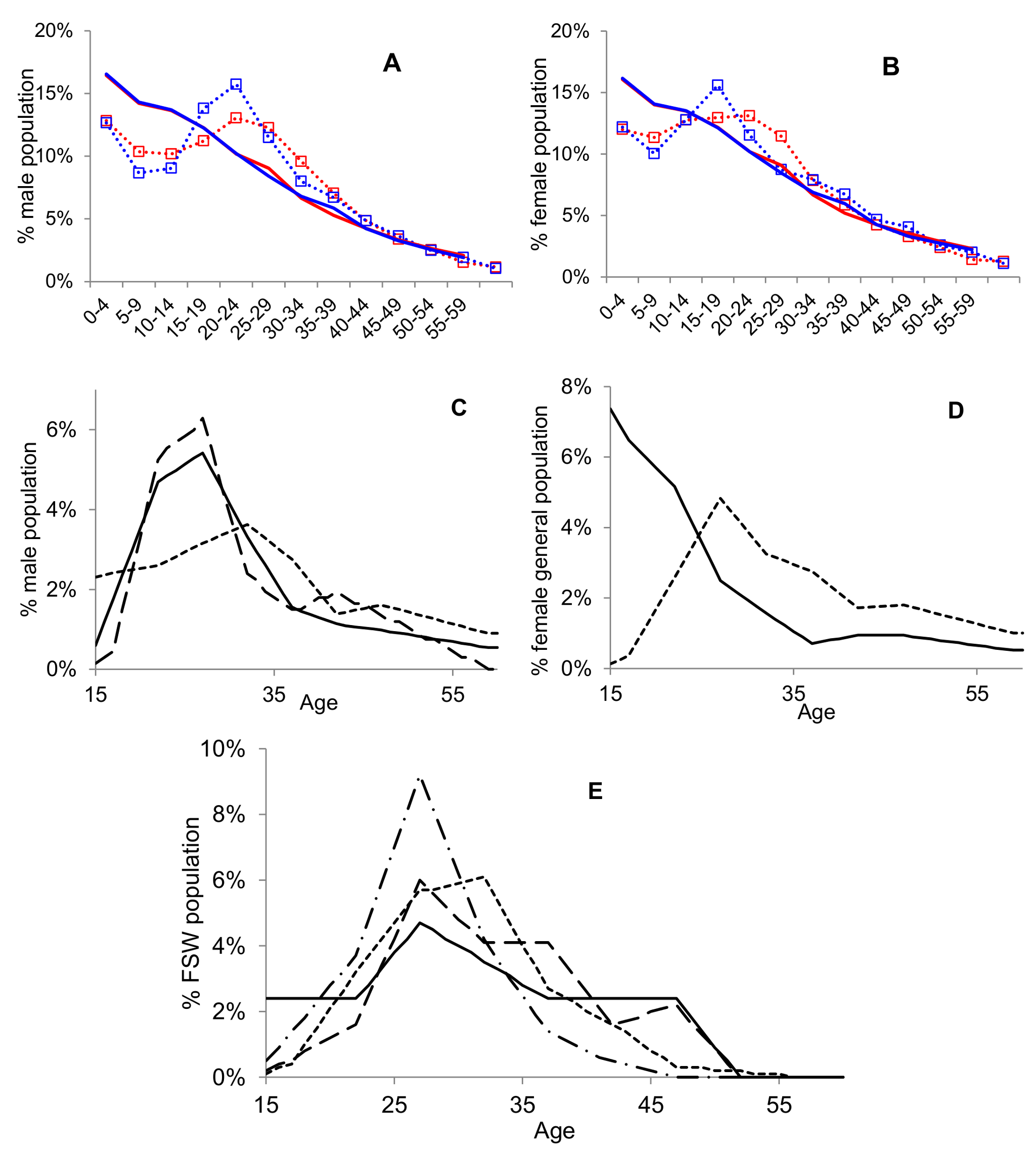

Supplement: Figure S4 — Initial population age distributions. Data (dotted line) and model projections (solid line) of population age distributions by 5 year age group for A) males and B) females for years 2002 (blue) and 2012 (red); and initial age distributions of each of the risk groups in the model: C) low risk (dotted line), moderate risk (solid line) and FSW clients (dashed line) males, D) low risk (dotted line) and moderate risk (dashed line) females in the general population and E) Beninese (solid line), Ghanaian (dotted line), Togolese (dashed line) and Nigerian (dot-dash line) FSWs. (TIF) [file pone.0102643.s004.tif]

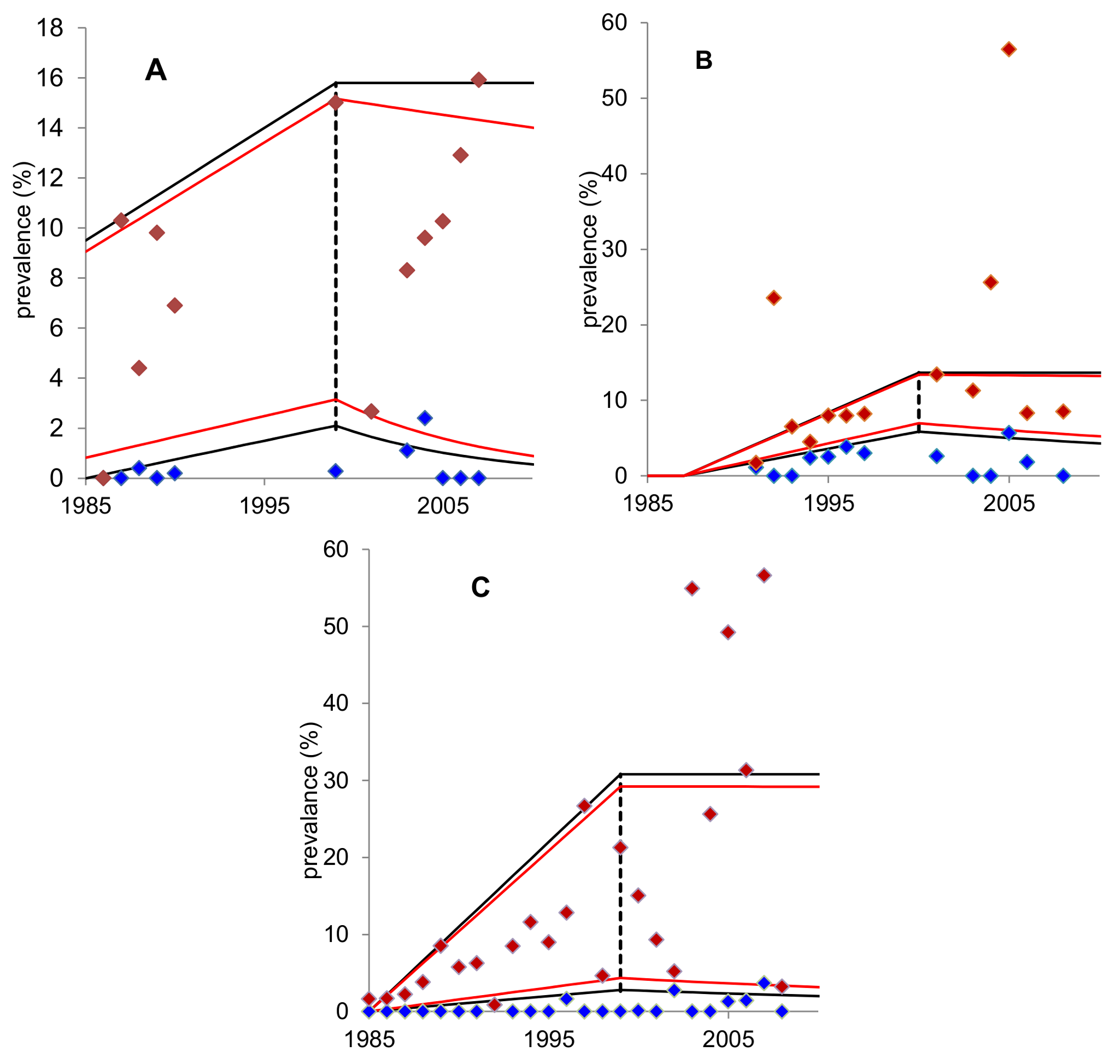

Supplement: Figure S6 — HIV prevalence of new entrant transnational FSWs. Limits (solid black lines) of ranges of HIV prevalence of new entrant FSWs in the model: A) Ghanaian, B) Togolese and C) Nigerian. For each nationality, LHS sampling provided a linear rate of increase in prevalence and a subsequent rate of exponential decline following the year of change from linear increase to exponential decline (dotted black line). Corresponding 5 and 95 percentiles for the posterior fits are shown (solid red lines), and also minimum (blue diamonds) and maximum (red diamonds) prevalence data reported for the source countries [21]. Data points include general population as well as FSW data based on the assumption that new entrant FSWs may not begin SW until they have left their home region. (TIF) [file pone.0102643.s006.tif]

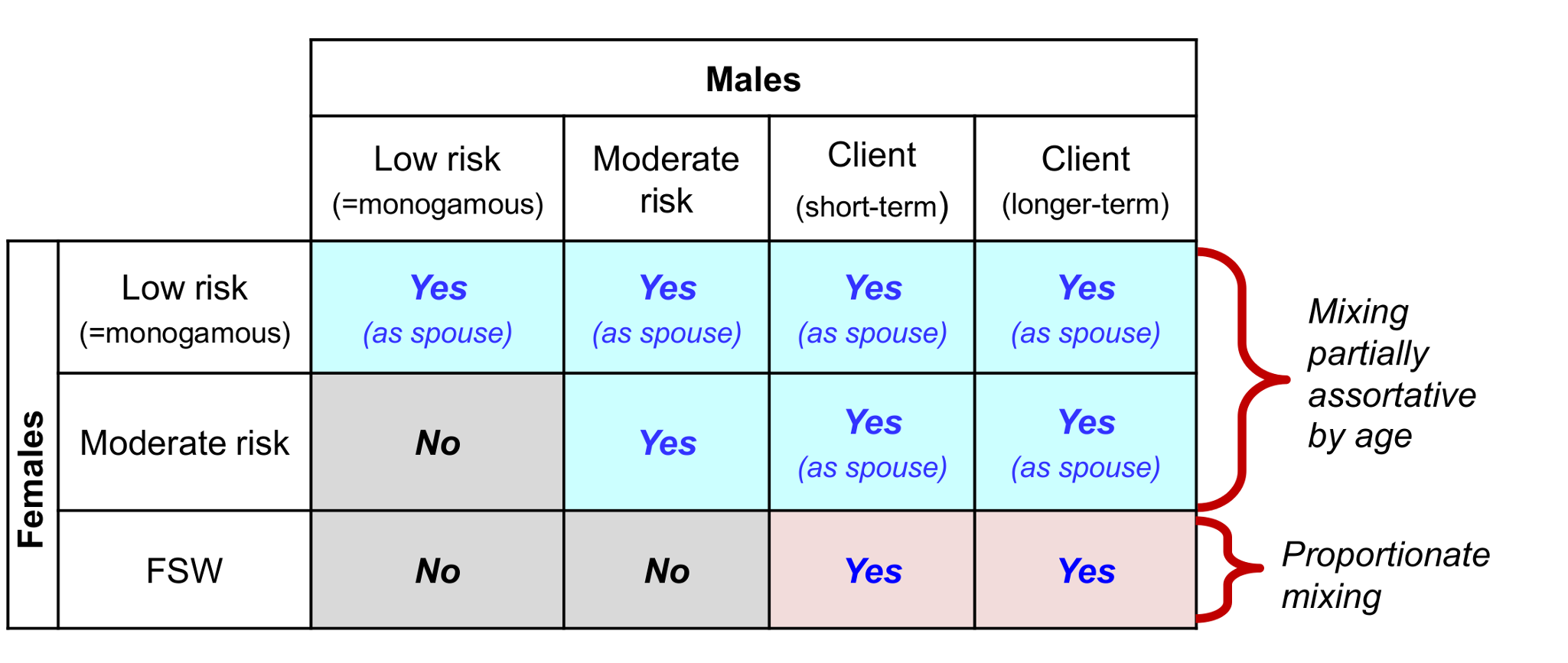

Supplement: Figure S7 — Mixing structure. Schematic diagram of pattern of contacts between risk groups which are allowed within the model. Note that males who are FSW clients may have non-commercial partnerships with other females whereas FSWs only have contacts with clients, some of whom may be “boy-friends”. (TIF) [file pone.0102643.s007.tif]

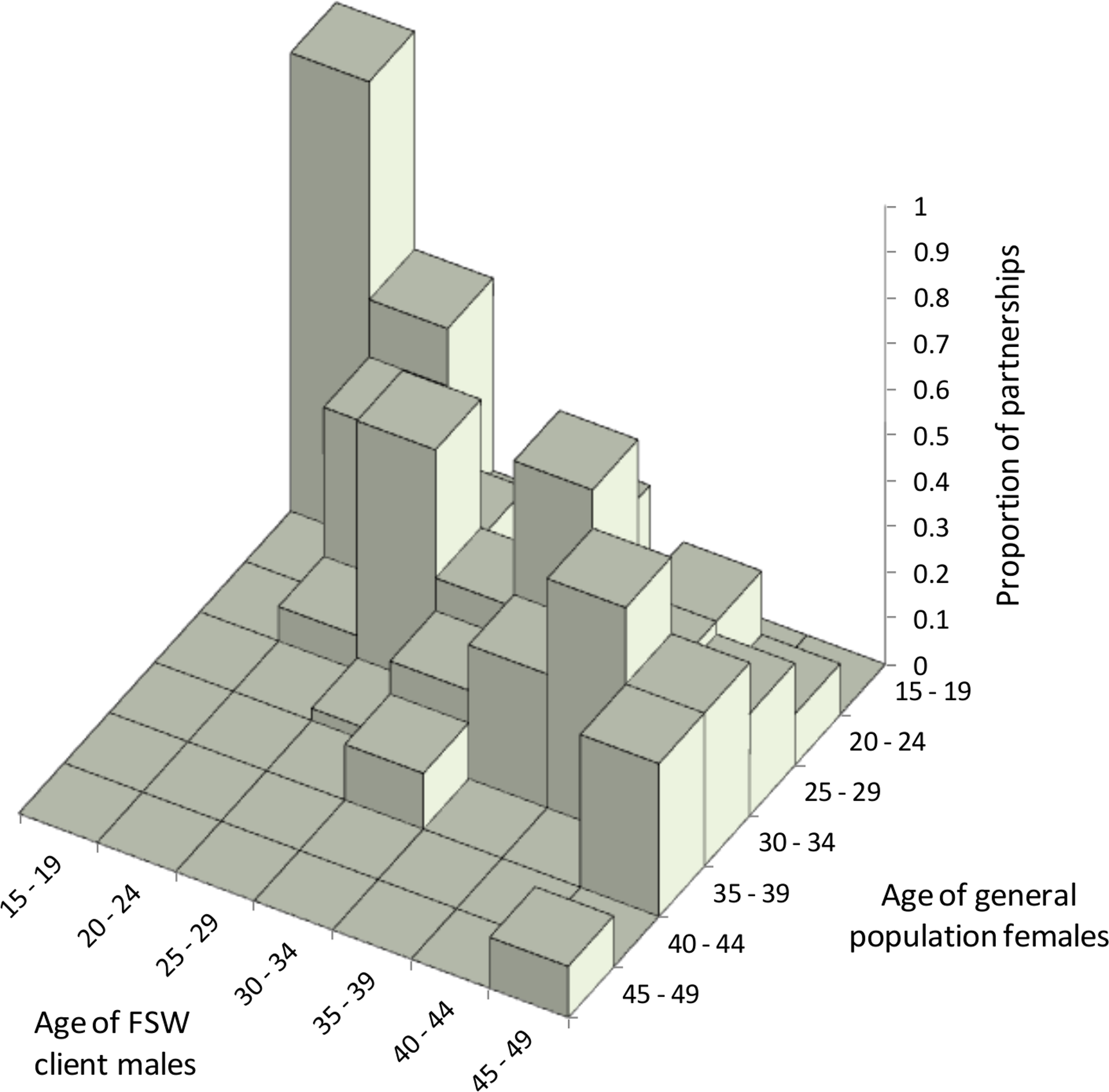

Supplement: Figure S8 — Contact patterns from data. Data illustrating the age distribution of partnerships with females in the general population of Cotonou reported by males who are also FSW clients (unpublished data from SIDA 1/2/3). (TIF) [file pone.0102643.s008.tif]

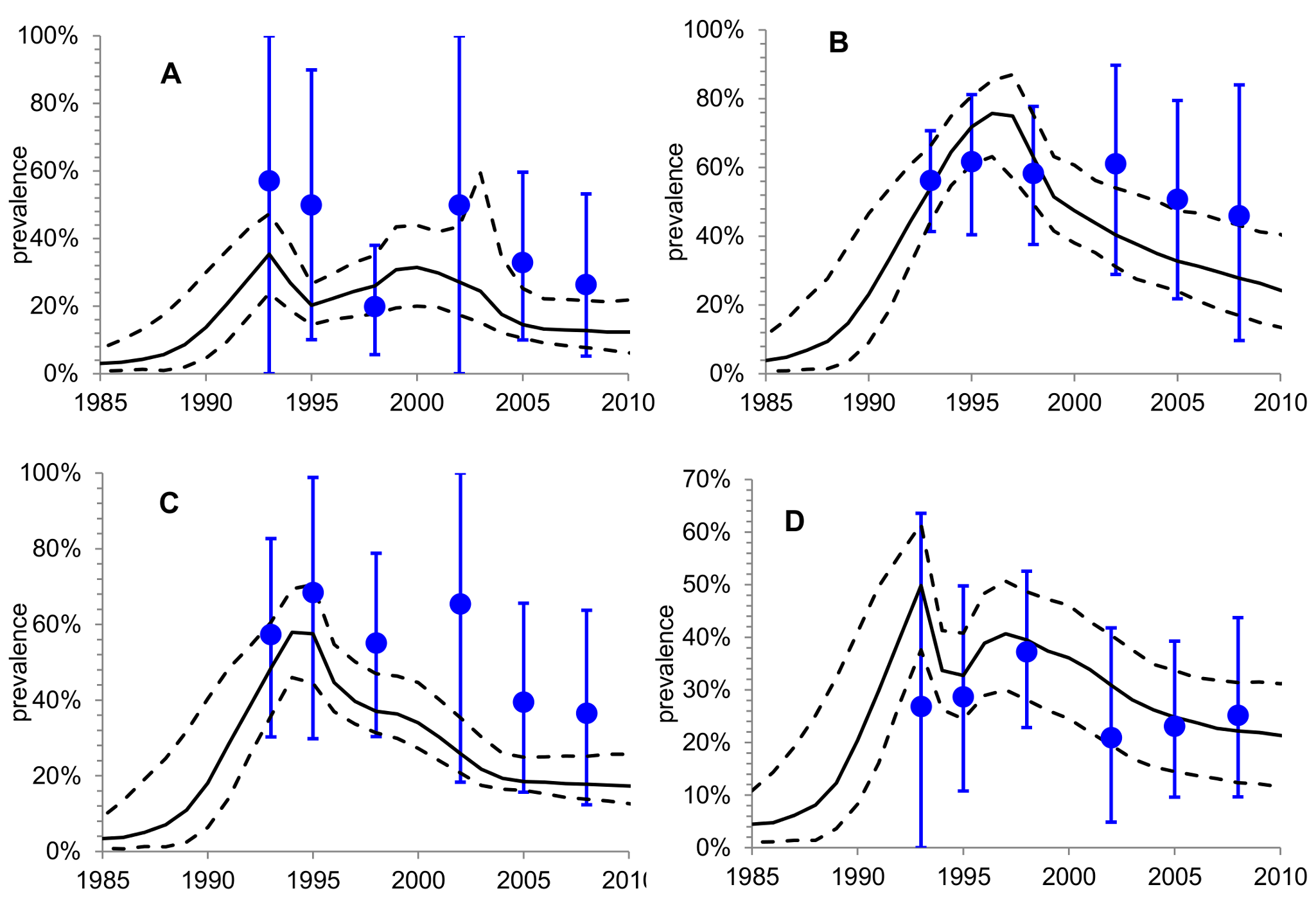

Supplement: Figure S9 — Model HIV prevalence results compared with data targets. HIV prevalence in different risk groups comparing model results and targets used in fitting the model: Results of each of the 472 model runs simultaneously fitting HIV prevalence predicted by the model to the 38 targets for the 4 FSW nationalities A) Benin, B) Ghana, C) Togo, D) Nigeria). Model results did not fit HIV prevalence for Beninese FSWs perfectly because the initially very low numbers of FSW reporting Beninese nationality resulted in very noisy trend estimates. (TIF) [file pone.0102643.s009.tif]

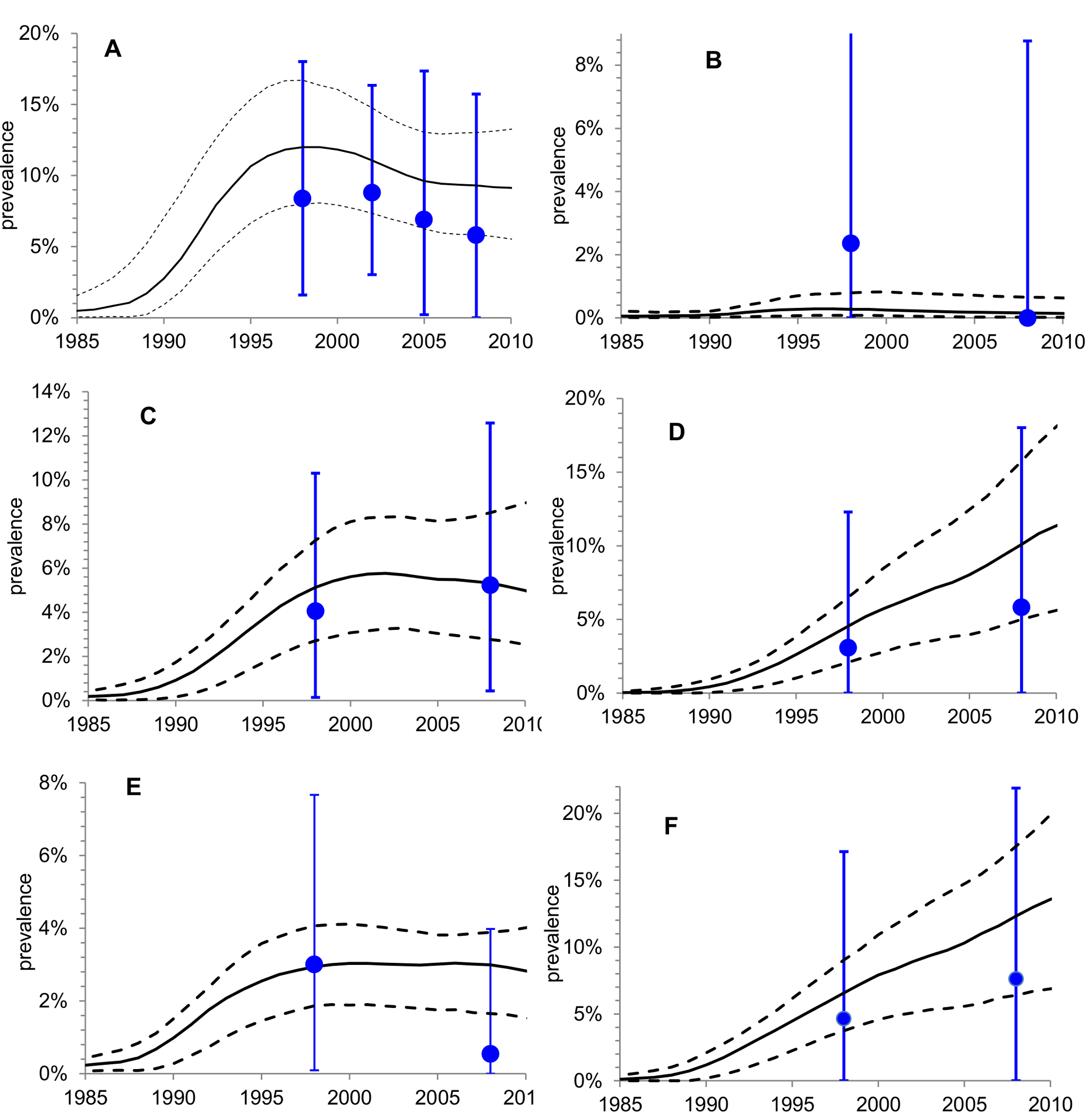

Supplement: Figure S10 — Model HIV prevalence results compared with data targets. HIV prevalence in different risk groups comparing model results and targets used in fitting the model: Results of each of the 472 model runs simultaneously fitting HIV prevalence predicted by the model to the 38 targets for A) clients, and general population females by age groups B) 15–19, C) 20–34, D)35–59, and males E) 15–34, F) 35–59. (TIF) [file pone.0102643.s010.tif]

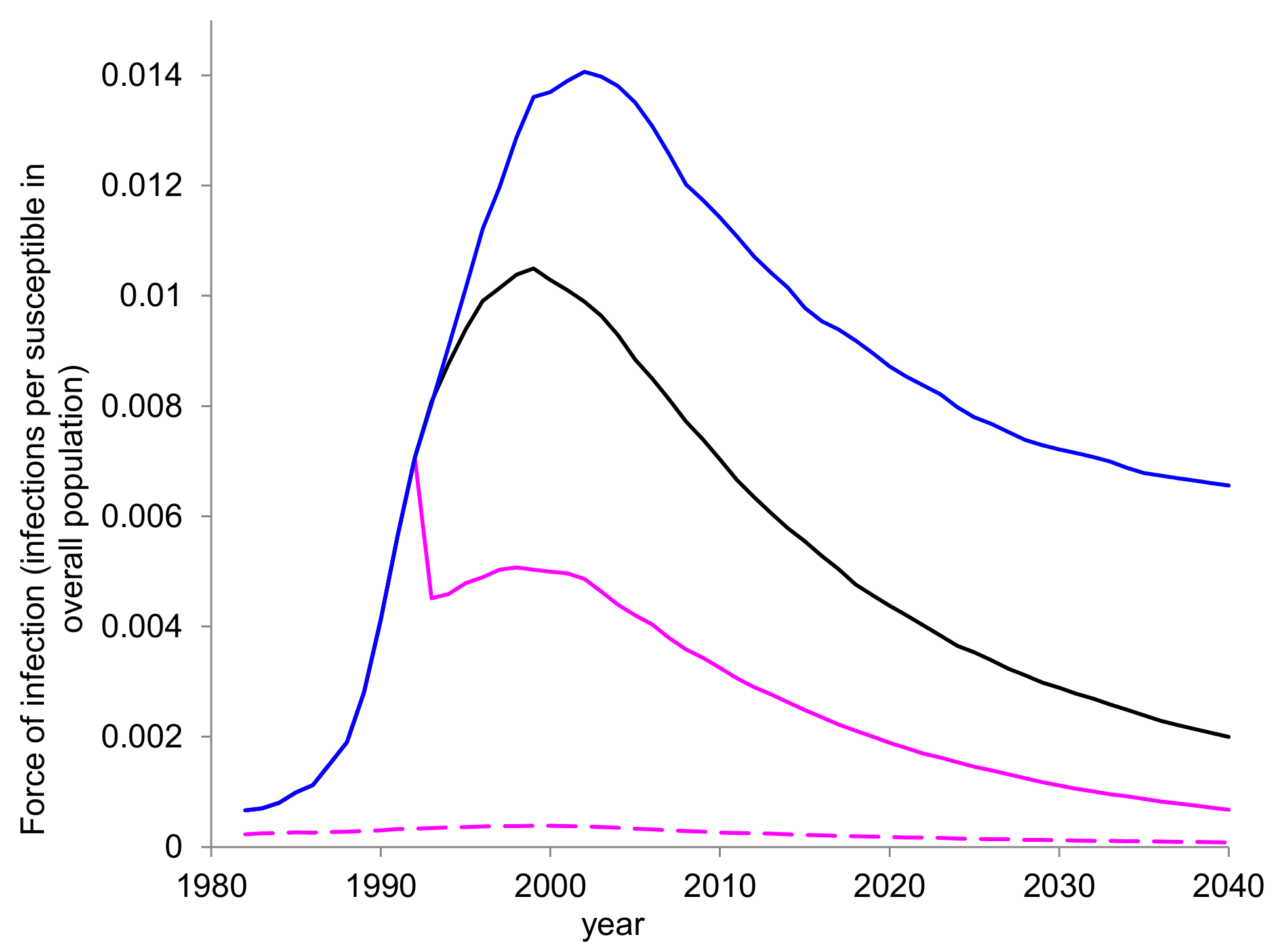

Supplement: Figure S11 — Force of HIV infection under different scenarios. Time trends in the annual rate of HIV per susceptible person in the general population: i) based on Project SIDA 1/2/3 FSW condom trends assumptions (black); ii) under counterfactual scenario CF-2 (blue); iii) assuming absolutely no transmission during commercial sex from 1993 (i.e. akin to a perfect intervention that would have started in 1993) (pink); and finally, iv) assuming absolutely no transmission during commercial sex since the start of the HIV epidemic (dashed pink). (TIF) [file pone.0102643.s011.tif]

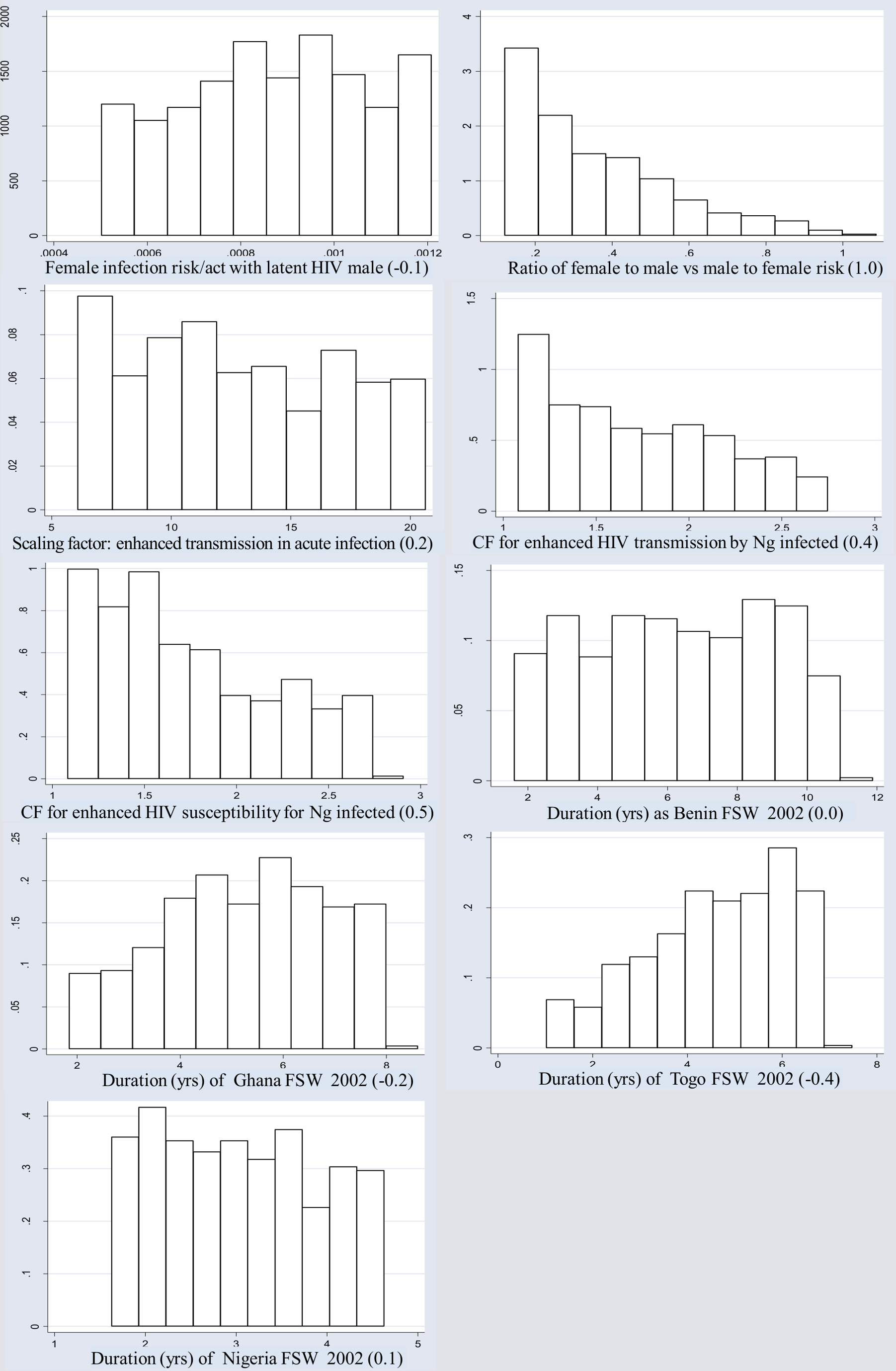

Supplement: Figure S12 — Selected distributions of posterior parameter ranges. Examples of the shapes of the distributions of the posterior parameter ranges shown in Table S1 (the associated skew values shown in the table are repeated in brackets after the title of each distribution). (TIF) [file pone.0102643.s012.tif]
