# Supplementary material for: Positive Impact of Increases in Condom Use among Female Sex Workers and Clients in a Medium HIV Prevalence Epidemic: Modelling Results from Project SIDA1/2/3 in Cotonou, Benin
Source: PLoS One. 2014 Jul 21;9(7):e102643. doi: 10.1371/journal.pone.0102643 (PMC4105482; doi:10.1371/journal.pone.0102643)
Supplement: Table S1 — Prior and posterior parameter ranges. S1a: Biological parameters. S1b: Intervention and treatment parameters. S1c: Behavioural parameters. S1d: Initial prevalence parameters. S1f: Migration. (DOC) [file pone.0102643.s013.doc]

**Table S1: Model parameters.** Ranges (uniformly distributed) of prior parameter values used in the modelling and posterior ranges of these parameters as used to generate model predictions (examples of distributions of these posterior values are shown in Figure S11). Posterior parameter ranges are shown to indicate the values used in the fitted set of model results and should not be considered estimates for these parameters. (NB. The skew values shown measure the degree of asymmetry, from -1.0 to1.0, shown by the posterior distributions with negative and positive skew values implying, respectively, longer tails at the lower and upper ends of the range of values).

**S1a Biological parameters.**

| **Parameter** |  | **Prior range** | | **Posterior range** | |  |  |
| --- | --- | --- | --- | --- | --- | --- | --- |
|  | **Units** | **Lower limit** | **Upper limit** | **5^th^ percentile** | **95^th^ percentile** | **Skew** | **Source/Reference** |
| **HIV progression rates:**  Primary to latent infection transition rate | /yr | 1.800 | 3.300 | 1.879 | 3.252 | -0.1 | Pickles, 2010  Grover, 2004  Kumarasamy, 2003 |
| Latent to pre-AIDS transition rate | /yr | 0.119 | 0.189 | 0.124 | 0.186 | -0.1 | Pickles, 2010  Grover, 2004  Kumarasamy, 2003 |
| Pre-AIDS to AIDS transition rate | /yr | 0.600 | 2.200 | 0.701 | 2.100 | 0.1 | Pickles, 2010  Grover, 2004  Kumarasamy, 2003 |
| AIDS mortality rate | /yr | 0.450 | 2.200 | 0.535 | 2.116 | 0.1 | Morgan, 2002 |
| **Duration of treatment prior to ARV failure/non-adherence (years)** | /yr | 0.180 | 0.396 | 0.191 | 0.386 | -0.1 | **†** |
| Female risk of *Ng* infection per unprotected act with infected male | /act/yr | 0.050 | 0.202 | 0.057 | 0.191 | 0.0 | Pickles, 2010  Holmes, 1970  Hooper, 1978 |
| Male risk of *Ng* infection per unprotected act with infected female | /act/yr | 0.050 | 0.207 | 0.056 | 0.196 | 0.3 | Pickles, 2010  Holmes, 1970  Hooper, 1978 |
| **Rate of recovery from treated *Ng***  Males | /yr | 4.110 | 24.988 | 4.981 | 24.106 | -0.1 | Pickles, 2010  Korenromp, 2002 |
| Females | /yr | 4.478 | 21.905 | 5.586 | 20.949 | 0.0 | Pickles, 2010  Korenromp, 2002 |
| **Rate of recovery from untreated *Ng***  Males | /yr | 2.302 | 6.409 | 2.602 | 6.282 | -0.2 | Pickles, 2010  Korenromp, 2002 |
| Females | /yr | 0.983 | 6.266 | 1.507 | 6.112 | 0.2 | Pickles, 2010  Korenromp, 2002 |
| **Proportion of *Ng* infected showing symptoms and potentially seeking treatment** |  |  |  |  |  |  |  |
| Males | - | 0.405 | 0.715 | 0.417 | 0.702 | 0.0 | Pickles, 2010 |
| Females | - | 0.180 | 0.330 | 0.185 | 0.320 | 0.1 | Pickles, 2010 |
| Cofactor for enhanced HIV transmission by those infected with gonorrhoea | **-** | 1.080 | 2.750 | 1.1276698 | 2.555 | 0.4 | Pickles, 2010  Korenromp, 2002  Rottingen, 2001 |
| Cofactor for enhanced HIV susceptibility for those infected with gonorrhoea | **-** | 1.080 | 2.750 | 1.150 | 2.639 | 0.5 | Pickles, 2010  Korenromp, 2002  Rottingen, 2001 |
| Male to female risk HIV transmission probability per unprotected sex act - latent stage of HIV infection | /act/yr | 0.000500 | 0.001200 | 0.000539 | 0.001172 | -0.1 | Pickles, 2010  Boily, 2009 |
| Ratio of female to male vs male to female risk | - | 0.120 | 1.00 | 0.137 | 0.771 | 1.0 | Pickles, 2010  Boily, 2009 |
| **Relative increase in transmission:**  during acute infection | /act/yr | 6.080 | 20.680 | 6.618 | 19.684 | 0.2 | Pickles, 2010  Boily, 2009 |
| during pre-AIDS stage | /act/yr | 4.050 | 13.090 | 4.530 | 12.672 | -0.2 | Pickles, 2010  Boily, 2009 |

†Assumption

**S1b Intervention and treatment parameters.**

| **Parameter** |  | **Prior range** | | **Posterior range** | |  |  |
| --- | --- | --- | --- | --- | --- | --- | --- |
|  | **Units** | **Lower limit** | **Upper limit** | **5^th^ percentile** | **95^th^ percentile** | **Skew** | **Source/Reference** |
| **Proportion of *Ng* cases treated at specified time point** |  |  |  |  |  |  |  |
| Non-client males 1986 | - | 0.176 | 0.301 | 0.182 | 0.295 | -0.1 | SIDA1/2/3 |
| Clients 1986 | - | 0.186 | 0.304 | 0.192 | 0.299 | 0.0 | SIDA1/2/3 |
| Clients 2002 | - | 0.361 | 0.527 | 0.368 | 0.519 | 0.1 | SIDA1/2/3 |
| Clients 2007 | - | 0.325 | 0.474 | 0.331 | 0.466 | 0.0 | SIDA1/2/3 |
| Non-FSW females 1986 | - | 0.083 | 0.144 | 0.085 | 0.141 | 0.0 | SIDA1/2/3 |
| FSW 1986 | - | 0.0110 | 0.0180 | 0.0112 | 0.0173 | 0.1 | SIDA1/2/3 |
| FSW 1993 | - | 0.371 | 0.564 | 0.379 | 0.554 | 0.0 | SIDA1/2/3 |
| FSW 2005 | - | 0.674 | 0.936 | 0.693 | 0.921 | -0.1 | SIDA1/2/3 |
| Condom efficacy against HIV | - | 0.744 | 1.000 | 0.752 | 0.974 | 0.5 | Pickles, 2010 |
| Condom efficacy against *Ng* | - | 0.545 | 0.880 | 0.563 | 0.854 | 0.1 | Pickles, 2010 |
| **Condom use (% acts) at specified time point** |  |  |  |  |  |  |  |
| FSW condom use in 1989 as proportion of 1993 use | - | 0.000 | 0.550 | 0.023 | 0.520 | 0.1 | † |
| Benin FSW in 1993 | % | 18.00 | 33.00 | 18.66 | 32.18 | 0.0 | SIDA1/2/3 |
| Benin FSW in 1998 | % | 40.00 | 72.90 | 41.51 | 71.23 | 0.0 | SIDA1/2/3 |
| Benin FSW in 2002 | % | 61.40 | 100.00 | 63.20 | 97.91 | 0.1 | SIDA1/2/3 |
| Benin FSW in 2005 | % | 61.20 | 100.00 | 62.97 | 97.48 | 0.2 | SIDA1/2/3 |

**S1b** **Intervention and treatment parameters (continued)**

| **Parameter** |  | **Prior range** | | **Posterior range** | |  |  |
| --- | --- | --- | --- | --- | --- | --- | --- |
|  | **Units** | **Lower limit** | **Upper limit** | **5^th^ percentile** | **95^th^ percentile** | **Skew** | **Source/Reference** |
| Benin FSW in 2008 | % | 69.60 | 100.00 | 71.30 | 98.58 | 0.1 | SIDA1/2/3 |
| Ghana FSW in 1993 | % | 18.00 | 33.00 | 19.00 | 32.29 | 0.0 | SIDA1/2/3 |
| Ghana FSW in 1998 | % | 40.00 | 72.90 | 41.70 | 71.91 | 0.0 | SIDA1/2/3 |
| Ghana FSW in 2002 | % | 72.40 | 100.00 | 73.27 | 98.36 | 0.1 | SIDA1/2/3 |
| Ghana FSW in 2005 | % | 71.50 | 100.00 | 72.75 | 98.41 | 0.0 | SIDA1/2/3 |
| Ghana FSW in 2008 | % | 68.90 | 100.00 | 70.80 | 98.79 | -0.1 | SIDA1/2/3 |
| Togo FSW in 1993 | % | 18.00 | 33.00 | 18.59 | 31.92 | 0.1 | SIDA1/2/3 |
| Togo FSW in 1998 | % | 40.00 | 72.90 | 41.42 | 71.71 | 0.1 | SIDA1/2/3 |
| Togo FSW in 2002 | % | 68.70 | 100.00 | 69.89 | 97.73 | 0.1 | SIDA1/2/3 |
| Togo FSW in 2005 | % | 67.30 | 100.00 | 68.61 | 96.99 | 0.1 | SIDA1/2/3 |
| Togo FSW in 2008 | % | 69.00 | 100.00 | 70.25 | 98.44 | 0.1 | SIDA1/2/3 |
| Nigeria FSW in 1993 | % | 18.00 | 33.00 | 18.89 | 32.41 | 0.0 | SIDA1/2/3 |
| Nigeria FSW in 1998 | % | 40.00 | 72.90 | 42.08 | 71.26 | 0.1 | SIDA1/2/3 |
| Nigeria FSW in 2002 | % | 86.40 | 100.00 | 87.31 | 99.47 | 0.0 | SIDA1/2/3 |
| Nigeria FSW in 2005 | % | 82.20 | 100.00 | 82.90 | 98.79 | 0.0 | SIDA1/2/3 |
| Nigeria FSW in 2008 | % | 83.10 | 100.00 | 84.21 | 99.26 | 0.0 | SIDA1/2/3 |
| Short-term clients in 1993 | % | 18.00 | 33.00 | 18.65 | 32.56 | 0.0 | SIDA1/2/3 |
| Long-term clients in 1993 | % | 18.00 | 33.00 | 18.71 | 32.51 | 0.0 | SIDA1/2/3 |
| Low risk males with low risk females 1998 | % | 1.16 | 12.18 | 1.74 | 11.44 | 0.0 | SIDA1/2/3 |
| Low risk females with moderate risk males 1998 | % | 1.16 | 12.18 | 1.69 | 11.60 | 0.0 | SIDA1/2/3 |
| Low risk females with moderate risk males 2008 | % | 1.16 | 12.18 | 1.64 | 11.69 | 0.0 | SIDA1/2/3 |
| Low risk females with partners who are short-term FSW clients 1998 | % | 1.16 | 12.18 | 1.75 | 11.60 | 0.1 | SIDA1/2/3 |
| Low risk females with partners who are short-term FSW clients 2008 | % | 1.16 | 12.18 | 1.77 | 11.52 | -0.1 | SIDA1/2/3 |
| Low risk females with partners who are long-term FSW clients 1998 | % | 1.16 | 12.18 | 1.60 | 11.72 | 0.1 | SIDA1/2/3 |
| Low risk females with partners who are long-term FSW clients 2002 | % | 8.00 | 21.84 | 8.63 | 21.28 | 0.1 | SIDA1/2/3 |
| Low risk females with partners who are long-term FSW clients 2005 | % | 16.20 | 41.28 | 17.33 | 39.54 | 0.0 | SIDA1/2/3 |
| Low risk females with partners who are long-term FSW clients 2008 | % | 28.40 | 59.52 | 29.69 | 58.26 | 0.0 | SIDA1/2/3 |
| Moderate risk females with low risk males 1998 | % | 1.16 | 12.18 | 1.69 | 11.64 | 0.0 | SIDA1/2/3 |
| Moderate risk females with moderate risk males 1998 | % | 8.50 | 36.72 | 9.61 | 35.44 | 0.0 | SIDA1/2/3 |
| Moderate risk females with moderate risk males 2008 | % | 29.20 | 69.24 | 31.48 | 66.82 | -0.1 | SIDA1/2/3 |

**S1b** **Intervention and treatment parameters (continued)**

| **Parameter** |  | **Prior range** | | **Posterior range** | |  |  |
| --- | --- | --- | --- | --- | --- | --- | --- |
|  | **Units** | **Lower limit** | **Upper limit** | **5^th^ percentile** | **95^th^ percentile** | **Skew** | **Source/Reference** |
| Moderate risk females with partners who are short-term FSW clients 1998 | % | 8.50 | 36.72 | 10.10 | 35.56 | -0.1 | SIDA1/2/3 |
| Moderate risk females with partners who are short-term FSW clients 2008 | % | 29.20 | 69.24 | 31.66 | 67.55 | 0.0 | SIDA1/2/3 |
| Moderate risk females with partners who are long-term FSW clients 1998 | % | 8.50 | 36.72 | 10.58 | 35.51 | -0.2 | SIDA1/2/3 |
| Moderate risk females with partners who are long-term FSW clients 2002 | % | 29.80 | 62.88 | 31.93 | 60.99 | 0.1 | SIDA1/2/3 |
| Moderate risk females with partners who are long-term FSW clients 2005 | % | 64.80 | 100.00 | 66.71 | 98.24 | 0.0 | SIDA1/2/3 |
| Moderate risk females with partners who are long-term FSW clients 2008 | % | 70.10 | 100.00 | 71.20 | 97.98 | 0.1 | SIDA1/2/3 |
| **Anti-retroviral treatment at specified time point** |  |  |  |  |  |  |  |
| 2004 | % | 4.50 | 11.00 | 4.84 | 10.72 | 0.0 | SIDA1/2/3 |
| 2006 | % | 22.50 | 68.20 | 24.65 | 66.37 | 0.0 | SIDA1/2/3 |
| 2009 | % | 40.50 | 96.80 | 43.63 | 94.41 | 0.0 | SIDA1/2/3 |

† Assumption

**S1c** **Behavioural parameters.**

| **Parameter** |  | **Prior range** | | **Posterior range** | |  |  |
| --- | --- | --- | --- | --- | --- | --- | --- |
|  | **Units** | **Lower limit** | **Upper limit** | **5^th^ percentile** | **95^th^ percentile** | **Skew** | **Source/Reference** |
| Degree of assortative mixing by age males with non-FSW females | - | 0.090 | 1.000 | 0.203 | 0.968 | -0.5 | † |
| Acts per short-term client contact with FSW | - | 0.900 | 3.300 | 1.010 | 3.145 | 0.2 | SIDA1/2/3 |
| Acts per long-term client contact with FSW | - | 0.675 | 2.547 | 0.797 | 2.406 | 0.1 | SIDA1/2/3 |
| Proportion of females becoming FSW who have been low risk/those ceasing FSW who become low risk | - | 0.000 | 1.000 | 0.050 | 0.949 | -0.2 | † |
| Proportion of non-client males who are low risk at debut | - | 0.173 | 0.770 | 0.209 | 0.753 | -0.1 | SIDA1/2/3 |
| Proportion of non-FSW females who are low risk at debut | - | 0.020 | 0.880 | 0.057 | 0.842 | 0.1 | SIDA1/2/3 |
| Proportion of females becoming FSW at debut | - | 0.00000 | 0.00019 | 0.00001 | 0.00018 | 0.1 | SIDA1/2/3 |
| Proportion of males becoming clients at debut | - | 0.090 | 0.330 | 0.113 | 0.319 | -0.1 | SIDA1/2/3 |
| Proportion of clients being "long-term" clients | - | 0.072 | 0.605 | 0.100 | 0.583 | 0.0 | SIDA1/2/3 |
| **Calculating weighted means of male-female partnerships** |  |  |  |  |  |  |  |
| Weighting of partnerships offered by non-FSW females | - | 0.714 | 1.000 | 0.937 | 0.998 | -0.8 | † |

**S1c** **Behavioural parameters (continued)**

| **Parameter** |  | **Prior range** | | **Posterior range** | |  |  |
| --- | --- | --- | --- | --- | --- | --- | --- |
|  | **Units** | **Lower limit** | **Upper limit** | **5^th^ percentile** | **95^th^ percentile** | **Skew** | **Source/Reference** |
| Weighting of partnerships offered by FSW | - | 0.000 | 0.605 | 0.030 | 0.565 | 0.1 | † |
| Scaling factor for acts per contact when converting risk per act to risk per contact in Ng sub-model | - | 0.105 | 0.702 | 0.118 | 0.664 | 0.5 | † |
| **Contact rates according to age group** |  |  |  |  |  |  |  |
| *Benin FSW* |  |  |  |  |  |  |  |
| 15-19 | ptnrs/yr | 56.7 | 666.1 | 80.3 | 635.7 | -0.1 | SIDA1/2/3 |
| 20-24 | ptnrs/yr | 311.0 | 756.8 | 323.0 | 727.9 | -0.1 | SIDA1/2/3 |
| 25-29 | ptnrs/yr | 342.7 | 819.0 | 364.7 | 788.9 | 0.0 | SIDA1/2/3 |
| 30-34 | ptnrs/yr | 378.9 | 1048.9 | 423.5 | 1018.9 | 0.0 | SIDA1/2/3 |
| 35-39 | ptnrs/yr | 447.3 | 1372.9 | 490.3 | 1329.6 | 0.1 | SIDA1/2/3 |
| 40-44 | ptnrs/yr | 131.9 | 1028.6 | 167.7 | 977.4 | 0.0 | SIDA1/2/3 |
| 45-59 | ptnrs/yr | 0.0 | 2899.0 | 119.1 | 2694.0 | 0.1 | SIDA1/2/3 |
| *Ghana FSW* |  |  |  |  |  |  |  |
| 15-19 | ptnrs/yr | 0.0 | 2662.2 | 176.9 | 2510.0 | -0.1 | SIDA1/2/3 |
| 20-24 | ptnrs/yr | 612.8 | 1217.1 | 648.5 | 1184.1 | 0.0 | SIDA1/2/3 |
| 25-29 | ptnrs/yr | 854.2 | 1420.6 | 881.6 | 1404.9 | 0.1 | SIDA1/2/3 |
| 30-34 | ptnrs/yr | 900.8 | 1487.4 | 928.7 | 1465.7 | -0.1 | SIDA1/2/3 |
| 35-39 | ptnrs/yr | 724.4 | 1286.5 | 752.1 | 1259.9 | -0.1 | SIDA1/2/3 |
| 40-44 | ptnrs/yr | 976.4 | 1750.3 | 1012.4 | 1722.1 | 0.0 | SIDA1/2/3 |
| 45-59 | ptnrs/yr | 317.6 | 2328.8 | 410.4 | 2170.1 | 0.0 | SIDA1/2/3 |
| *Togo FSW* |  |  |  |  |  |  |  |
| 15-19 | ptnrs/yr | 147.9 | 590.1 | 170.1 | 574.7 | -0.1 | SIDA1/2/3 |
| 20-24 | ptnrs/yr | 423.0 | 928.0 | 443.8 | 902.7 | .00 | SIDA1/2/3 |
| 25-29 | ptnrs/yr | 465.0 | 984.8 | 491.7 | 958.3 | -0.1 | SIDA1/2/3 |
| 30-34 | ptnrs/yr | 545.1 | 1112.1 | 587.7 | 1085.2 | 0.0 | SIDA1/2/3 |
| 35-39 | ptnrs/yr | 644.4 | 1432.3 | 704.4 | 1391.5 | -0.2 | SIDA1/2/3 |
| 40-44 | ptnrs/yr | 707.9 | 1698.6 | 771.3 | 1655.2 | -0.2 | SIDA1/2/3 |
| 45-59 | ptnrs/yr | 555.5 | 1682.4 | 628.3 | 1623.6 | 0.0 | SIDA1/2/3 |
| *Nigeria FSW* |  |  |  |  |  |  |  |
| 15-19 | ptnrs/yr | 796.6 | 2305.3 | 867.4 | 2231.9 | 0.1 | SIDA1/2/3 |
| 20-24 | ptnrs/yr | 790.7 | 1332.4 | 825.8 | 1299.1 | 0.1 | SIDA1/2/3 |

**S1c** **Behavioural parameters (continued)**

| **Parameter** |  | **Prior range** | | **Posterior range** | |  |  |
| --- | --- | --- | --- | --- | --- | --- | --- |
|  | **Units** | **Lower limit** | **Upper limit** | **5^th^ percentile** | **95^th^ percentile** | **Skew** | **Source/Reference** |
| 25-29 | ptnrs/yr | 730.2 | 1235.1 | 748.4 | 1199.0 | 0.0 | SIDA1/2/3 |
| 30-34 | ptnrs/yr | 585.6 | 1216.8 | 607.1 | 1167.4 | 0.0 | SIDA1/2/3 |
| 35-39 | ptnrs/yr | 758.6 | 1527.2 | 796.3 | 1485.4 | 0.1 | SIDA1/2/3 |
| 40-44 | ptnrs/yr | 560.2 | 1598.5 | 593.6 | 1532.5 | 0.0 | SIDA1/2/3 |
| 45-59 | ptnrs/yr | 781.7 | 2467.4 | 839.3 | 2381.1 | 0.0 | SIDA1/2/3 |
| *Moderate risk females* |  |  |  |  |  |  |  |
| 15-19 | ptnrs/yr | 0.070 | 0.260 | 0.075 | 0.229 | -0.1 | SIDA1/2/3 |
| 20-24 | ptnrs/yr | 0.210 | 0.530 | 0.220 | 0.479 | -0.2 | SIDA1/2/3 |
| 25-29 | ptnrs/yr | 0.110 | 0.470 | 0.118 | 0.395 | -0.1 | SIDA1/2/3 |
| 30-34 | ptnrs/yr | 0.110 | 0.640 | 0.113 | 0.464 | 0.0 | SIDA1/2/3 |
| 35-39 | ptnrs/yr | 0.040 | 0.430 | 0.044 | 0.299 | 0.0 | SIDA1/2/3 |
| 40-59 | ptnrs/yr | 0.040 | 0.330 | 0.044 | 0.232 | -0.1 | SIDA1/2/3 |
| *Low risk females* | ptnrs/yr |  |  |  |  |  |  |
| 15-19 | ptnrs/yr | 0.070 | 0.260 | 0.076 | 0.226 | -0.1 | SIDA1/2/3 |
| 20-24 | ptnrs/yr | 0.210 | 0.530 | 0.220 | 0.477 | -0.3 | SIDA1/2/3 |
| 25-29 | ptnrs/yr | 0.110 | 0.470 | 0.117 | 0.379 | -0.2 | SIDA1/2/3 |
| 30-34 | ptnrs/yr | 0.110 | 0.640 | 0.112 | 0.421 | -0.2 | SIDA1/2/3 |
| 35-39 | ptnrs/yr | 0.040 | 0.430 | 0.044 | 0.255 | 0.0 | SIDA1/2/3 |
| 40-59 | ptnrs/yr | 0.040 | 0.330 | 0.043 | 0.186 | -0.1 | SIDA1/2/3 |
|  |  |  |  |  |  |  |  |
| *Moderate risk males* |  |  |  |  |  |  |  |
| 15-19 | ptnrs/yr | 1.09 | 1.84 | 1.11 | 1.80 | -0.1 | SIDA1/2/3 |
| 20-24 | ptnrs/yr | 1.24 | 2.10 | 1.26 | 2.01 | 0.0 | SIDA1/2/3 |
| 25-29 | ptnrs/yr | 1.32 | 2.90 | 1.36 | 2.73 | 0.1 | SIDA1/2/3 |
| 30-34 | ptnrs/yr | 1.44 | 2.72 | 1.48 | 2.55 | 0.0 | SIDA1/2/3 |
| 35-39 | ptnrs/yr | 1.60 | 2.56 | 1.63 | 2.49 | -0.1 | SIDA1/2/3 |
| 40-44 | ptnrs/yr | 1.86 | 3.17 | 1.89 | 3.08 | 0.1 | SIDA1/2/3 |
| 45-59 | ptnrs/yr | 1.35 | 2.25 | 1.38 | 2.19 | -0.1 | SIDA1/2/3 |
| *Low risk males* |  |  |  |  |  |  |  |
| 15-19 | ptnrs/yr | 0.010 | 0.140 | 0.014 | 0.091 | -0.1 | SIDA1/2/3 |

**S1c** **Behavioural parameters (continued)**

| **Parameter** |  | **Prior range** | | **Posterior range** | |  |  |
| --- | --- | --- | --- | --- | --- | --- | --- |
|  | **Units** | **Lower limit** | **Upper limit** | **5^th^ percentile** | **95^th^ percentile** | **Skew** | **Source/Reference** |
| 20-24 | ptnrs/yr | 0.010 | 0.140 | 0.014 | 0.091 | 0.0 | SIDA1/2/3 |
| 25-29 | ptnrs/yr | 0.360 | 0.670 | 0.371 | 0.639 | 0.0 | SIDA1/2/3 |
| 30-34 | ptnrs/yr | 0.640 | 0.940 | 0.650 | 0.916 | 0.1 | SIDA1/2/3 |
| 35-39 | ptnrs/yr | 0.700 | 1.120 | 0.710 | 1.080 | 0.0 | SIDA1/2/3 |
| 40-44 | ptnrs/yr | 0.650 | 1.120 | 0.661 | 1.079 | 0.1 | SIDA1/2/3 |
| 45-49 | ptnrs/yr | 0.850 | 1.440 | 0.866 | 1.394 | 0.0 | SIDA1/2/3 |
| 50-59 | ptnrs/yr | 0.790 | 1.670 | 0.807 | 1.546 | 0.0 | SIDA1/2/3 |
| *Clients with non-FSW partners* |  |  |  |  |  |  |  |
| 15-19 | ptnrs/yr | 0.34 | 2.23 | 0.36 | 1.74 | -0.1 | SIDA1/2/3 |
| 20-24 | ptnrs/yr | 1.03 | 2.51 | 1.07 | 2.31 | 0.0 | SIDA1/2/3 |
| 25-29 | ptnrs/yr | 1.08 | 3.01 | 1.12 | 2.73 | -0.1 | SIDA1/2/3 |
| 30-34 | ptnrs/yr | 1.29 | 4.48 | 1.34 | 3.98 | -0.1 | SIDA1/2/3 |
| 35-39 | ptnrs/yr | 1.57 | 3.27 | 1.62 | 3.08 | -0.1 | SIDA1/2/3 |
| 40-44 | ptnrs/yr | 1.41 | 3.09 | 1.45 | 2.94 | 0.0 | SIDA1/2/3 |
| 45-59 | ptnrs/yr | 1.55 | 4.02 | 1.62 | 3.73 | 0.0 | SIDA1/2/3 |
| *Short-term clients with FSW* |  |  |  |  |  |  |  |
| 15-19 | ptnrs/yr | 7.60 | 16.97 | 8.00 | 16.35 | 0.0 | SIDA1/2/3 |
| 20-24 | ptnrs/yr | 8.33 | 14.23 | 8.71 | 13.97 | 0.0 | SIDA1/2/3 |
| 25-29 | ptnrs/yr | 9.50 | 16.55 | 9.80 | 16.20 | 0.0 | SIDA1/2/3 |
| 30-34 | ptnrs/yr | 8.49 | 16.95 | 9.01 | 16.58 | 0.0 | SIDA1/2/3 |
| 35-39 | ptnrs/yr | 7.65 | 19.34 | 8.31 | 18.63 | 0.0 | SIDA1/2/3 |
| 40-59 | ptnrs/yr | 8.41 | 19.34 | 8.86 | 18.89 | -0.1 | SIDA1/2/3 |
| *Long-term clients with FSW* |  |  |  |  |  |  |  |
| 15-19 | ptnrs/yr | 38.09 | 86.50 | 40.63 | 84.45 | 0.1 | SIDA1/2/3 |
| 20-24 | ptnrs/yr | 27.63 | 54.70 | 29.23 | 53.50 | 0.0 | SIDA1/2/3 |
| 25-29 | ptnrs/yr | 27.73 | 51.95 | 28.66 | 50.67 | 0.0 | SIDA1/2/3 |
| 30-34 | ptnrs/yr | 22.66 | 54.11 | 24.39 | 52.44 | 0.1 | SIDA1/2/3 |
| 35-39 | ptnrs/yr | 24.69 | 52.73 | 25.80 | 51.30 | 0.0 | SIDA1/2/3 |
| 40-59 | ptnrs/yr | 24.69 | 52.73 | 26.42 | 51.40 | 0.0 | SIDA1/2/3 |
| **Acts per partnership** |  |  |  |  |  |  |  |

**S1c** **Behavioural parameters (continued)**

| **Parameter** |  | **Prior range** | | **Posterior range** | |  |  |
| --- | --- | --- | --- | --- | --- | --- | --- |
|  | **Units** | **Lower limit** | **Upper limit** | **5^th^ percentile** | **95^th^ percentile** | **Skew** | **Source/Reference** |
| Low risk males with low risk females | acts/ptnr | 35.32 | 48.40 | 35.93 | 47.75 | -0.1 | SIDA1/2/3‡ |
| Moderate risk males with low risk females | acts/ptnr | 27.01 | 44.15 | 27.74 | 43.00 | 0.0 | SIDA1/2/3‡ |
| Moderate risk males with moderate risk females | acts/ptnr | 31.04 | 56.30 | 32.16 | 54.90 | 0.1 | SIDA1/2/3‡ |
| Low risk females with males who are FSW clients | acts/ptnr | 25.27 | 64.45 | 27.12 | 62.33 | 0.0 | SIDA1/2/3‡ |

† Ranges based on assumptions

‡Ranges indirectly derived from SIDA1/2/3 data

**S1d** **Initial prevalence parameters.** Ng prevalences were seeded prior to running the model to equilibrium (i.e. constant Ng prevalence), at which point which HIV was seeded into the population and the model run proper was started (NB Ranges for priors in this table were based upon assumptions about plausible values).

| **Parameter** |  | **Prior range** | | **Posterior range** | |  |  |
| --- | --- | --- | --- | --- | --- | --- | --- |
|  | **Units** | **Lower limit** | **Upper limit** | **5^th^ percentile** | **95^th^ percentile** | **Skew** | **Source/Reference** |
| **Initial Ng prevalences** |  |  |  |  |  |  |  |
| Low risk males | - | 0.001 | 0.021 | 0.002 | 0.020 | 0.0 | † |
| Moderate risk males | - | 0.001 | 0.021 | 0.002 | 0.020 | -0.1 | † |
| Clients | - | 0.032 | 0.129 | 0.036 | 0.123 | 0.0 | † |
| Low risk females | - | 0.005 | 0.032 | 0.007 | 0.030 | 0.0 | † |
| Moderate risk females | - | 0.005 | 0.031 | 0.006 | 0.030 | 0.0 | † |
| Benin FSW | - | 0.048 | 0.837 | 0.096 | 0.803 | 0.1 | † |
| Ghana FSW | - | 0.353 | 0.609 | 0.363 | 0.598 | 0.0 | † |
| Togo FSW | - | 0.213 | 0.556 | 0.228 | 0.538 | 0.0 | † |
| Nigeria FSW | - | 0.176 | 0.616 | 0.201 | 0.595 | 0.0 | † |
| **Initial HIV prevalences** |  |  |  |  |  |  |  |
| Benin FSW | - | 0.00450 | 0.05500 | 0.00674 | 0.05291 | 0.0 | † |
| Ghana FSW | - | 0.00450 | 0.05500 | 0.00723 | 0.05209 | 0.0 | † |
| Togo FSW | - | 0.00450 | 0.05500 | 0.00762 | 0.05224 | -0.1 | † |
| Nigeria FSW | - | 0.00450 | 0.05500 | 0.00665 | 0.05212 | -0.1 | † |
| Clients | - | 0.00000 | 0.02200 | 0.00131 | 0.02081 | 0.1 | † |
| Moderate risk males | - | 0.00000 | 0.00550 | 0.00020 | 0.00513 | 0.0 | † |
| Moderate risk females | - | 0.00000 | 0.00550 | 0.00031 | 0.00522 | 0.0 | † |
| **Adjustment to nominal HIV introduction year** |  | -2.500 | 4.900 | -1.669 | +4.585 | -0.3 | † |

**S1f** **Migration.** Parameters determining rates of flow into and out of FSW (inward and outward migration in the case of non-Beninese FSW)

| **Parameter** |  | **Prior range** | | **Posterior range** | |  |  |
| --- | --- | --- | --- | --- | --- | --- | --- |
|  | **Units** | **Lower limit** | **Upper limit** | **5^th^ percentile** | **95^th^ percentile** | **Skew** | **Source/Reference** |
| **Duration of FSW in Cotonou** |  |  |  |  |  |  |  |
| Benin FSW 2002 | yrs | 1.60 | 10.96 | 2.18 | 10.41 | 0.0 | SIDA1/2/3 |
| Ghana FSW 2002 | yrs | 1.79 | 8.02 | 2.41 | 7.75 | -0.2 | SIDA1/2/3 |
| Togo FSW 2002 | yrs | 0.98 | 6.89 | 1.82 | 6.67 | -0.4 | SIDA1/2/3 |
| Nigeria FSW 2002 | yrs | 1.62 | 4.63 | 1.72 | 4.50 | 0.1 | SIDA1/2/3 |
| Benin FSW 2005 | yrs | 1.08 | 4.69 | 1.41 | 4.51 | -0.1 | SIDA1/2/3 |
| Ghana FSW 2005 | yrs | 2.46 | 10.82 | 2.97 | 10.46 | 0.0 | SIDA1/2/3 |
| Togo FSW 2005 | yrs | 1.45 | 6.44 | 2.02 | 6.22 | -0.2 | SIDA1/2/3 |
| Nigeria FSW 2005 | yrs | 1.98 | 5.50 | 2.20 | 5.29 | -0.2 | SIDA1/2/3 |
| Inward FSW migration rate adjustment‡ | - | 0.90 | 1.10 | 0.91 | 1.09 | 0.1 | † |
| Outward FSW migration rate adjustment‡ | - | 0.90 | 1.10 | 0.91 | 1.09 | 0.1 | † |

† As data referred to duration of CSW in that location rather than migration specifically, adjustment of up to +/-10% was provided for during the fitting

‡ Correlation between these adjustments = -0.0336

**References**

Boily MC, Baggaley RF, Wang L, Masse B, White RG, Hayes RJ, Alary M. (2009) Heterosexual risk of HIV-1 infection per sexual act: systematic review and meta-analysis of observational studies. Lancet Infect Dis. 9(2): 118-29.

Grover G,Shivraj SO. (2004) Survival pattern of reported HIV infected individuals in the city of Delhi (India) .J Commun Dis 36:83e92

Holmes KK,Johnson DW, Trostle HJ. (1970) An estimate of the risk of men acquiring gonorrhea by sexual contact with infected females. Am J Epidemiol 91:170e4.

Hooper RR,Reynolds GH, Jones OG,et al. (1978) Cohort study of venereal disease. I: the risk of gonorrhea transmission from infected women to men. Am J Epidemiol 108:136e44.

Korenromp EL, Sudaryo MK, de Vlas SJ, Gray RH, Sewankambo NK, Serwadda D, Wawer MJ, Habbema JD (2002) What proportion of episodes of gonorrhoea and chlamydia becomes symptomatic? Int J STD AIDS. 13(2):91-101

Kumarasamy N,Solomon S, Flanigan TP,et al. (2003) Natural history of human immunodeficiency virus disease in southern India. Clin Infect Dis 36:79e85.

Morgan D, Mahe C, Mayanja B, Okongo JM, Lubega R, Whitworth JA (2002) HIV-1 infection in rural Africa: is there a difference in median time to AIDS and survival compared with that in industrialized countries? AIDS. 2002 16(4):597-603

Pickles M, Boily MC, Vickerman P, Lowndes CM, Moses S et al (2013) Assessment of the population-level effectiveness of the Avahan HIV-prevention programme in South India: a preplanned, causal-pathway-based modelling analysis. Lancet Global Health 1(5):e289-e299.

Rottingen JA,Cameron DW, Garnett GP. (2001) A systematic review of the epidemiologic interactions between classic sexually transmitted diseases and HIV: how much really is known? Sex Transm Dis 28:579e97
