# Supplementary material for: Positive Impact of Increases in Condom Use among Female Sex Workers and Clients in a Medium HIV Prevalence Epidemic: Modelling Results from Project SIDA1/2/3 in Cotonou, Benin
Source: PLoS One. 2014 Jul 21;9(7):e102643. doi: 10.1371/journal.pone.0102643 (PMC4105482; doi:10.1371/journal.pone.0102643)
Supplement: Table S2 — Demographic parameters. S2a: Estimated net rates of change in FSW populations per year by nationality. S2b: Demography. (DOC) [file pone.0102643.s014.doc]

**Table S2a: Estimated net rates of change per year** in FSW populations per year by nationality

| **Year** | **Beninese** | **Ghanaian** | **Togolese** | **Nigerian** |
| --- | --- | --- | --- | --- |
| 1993 | 1.35 | -0.23 | -0.21 | 0.84 |
| 1995 | 0.30 | -0.17 | 0.08 | 0.01 |
| 1998 | -0.23 | -0.01 | -0.02 | 0.09 |
| 2002 | 0.32 | -0.12 | 0.16 | -0.08 |
| 2005 | 0.06 | -0.08 | -0.01 | 0.00 |

NB 1. Linear interpolation was used to estimate rates between years specified in table.

2. The net change in numbers of Benin FSW was adjusted over time to ensure that the proportion of all FSW from Benin reflected estimates from SIDA1/2/3 data.

**Table S2b: Demography: Vital rates** (Source: US Census Bureau database[21])

| **Age group (yrs)** | **Mortality/yr** | | **Age specific fertility/yr** |
| --- | --- | --- | --- |
|  | *Male* | *Female* |  |
| 0-4 | 0.04189 | 0.0396 | - |
| 5-9 | 0.00339 | 0.00254 | - |
| 10-14 | 0.00151 | 0.00088 | - |
| 15-19 | 0.00066 | 0.0005 | 0.1243 |
| 20-24 | 0.00219 | 0.00158 | 0.2709 |
| 25-29 | 0.00333 | 0.00224 | 0.2919 |
| 30-34 | 0.0043 | 0.00246 | 0.2672 |
| 35-39 | 0.00567 | 0.00272 | 0.1932 |
| 40-44 | 0.00757 | 0.00316 | 0.1219 |
| 45-49 | 0.01052 | 0.0046 | 0.031 |
| 50-54 | 0.01508 | 0.00762 | - |
| 55-59 | 0.02321 | 0.01325 | - |
