# Supplementary material for: Positive Impact of Increases in Condom Use among Female Sex Workers and Clients in a Medium HIV Prevalence Epidemic: Modelling Results from Project SIDA1/2/3 in Cotonou, Benin
Source: PLoS One. 2014 Jul 21;9(7):e102643. doi: 10.1371/journal.pone.0102643 (PMC4105482; doi:10.1371/journal.pone.0102643)
Supplement: Table S3 — Correlations between PAFs and model parameters. (DOC) [file pone.0102643.s015.doc]

**Table S3. Correlations between PAFs and model parameters.** Pearson’s Correlation Coefficients (*r*) between the PAF (epidemic start-1993) and PAF(1993-2008) and all model parameters. Only the 5 parameters which were either statistically significant or had |r|>0.3 are shown in the table.

| **Parameter** | **PAF**  **(Epidemic start to 1993)** | | **PAF (1993-2008)** | |
| --- | --- | --- | --- | --- |
|  | ***(r)*** | ***p-value*** | ***(r)*** | ***p-value*** |
| Adjustment to nominal HIV introduction year† | 0.26 | *<0.001* | 0.51 | *<0.001* |
| Initial prevalence – moderate risk males | -0.48 | *<0.001* | -0.07 | *0.15* |
| Initial prevalence – moderate risk females | -0.32 | *<0.001* | -0.03 | *0.52* |
| Ratio of female to male vs male to female transmission probability | 0.39 | *<0.001* | 0.14 | *0.003* |
| Weighting of partnerships offered by non-FSW females†† | 0.26 | *<0.001* | 0.30 | *<0.001* |

†Range -2.5 to +4.9 years

††As numbers of partnerships by males must at all time points equal the number of partnerships by females in this heterosexual model, a simple procedure is used employing this weighting factor φ (see Text 1: Equations) to ensure that numbers of male and female partnerships balance as relative numbers of males and females change over time (a φ value of 0 represents a balance dominated by males and a value of 1 one dominated by females).
