# Supplementary material for: Positive Impact of Increases in Condom Use among Female Sex Workers and Clients in a Medium HIV Prevalence Epidemic: Modelling Results from Project SIDA1/2/3 in Cotonou, Benin
Source: PLoS One. 2014 Jul 21;9(7):e102643. doi: 10.1371/journal.pone.0102643 (PMC4105482; doi:10.1371/journal.pone.0102643)
Supplement: Text S1 — Model equations. (DOC) [file pone.0102643.s016.doc]

**Text S1: Model equations**

The model of population and HIV transmission dynamics is based on a system of partial differential equations (PDE), but encapsulates also, as a sub-model, a system of ordinary differential equations (ODE), incorporating the collapsed age structure of the PDE model, and representing gonorrhoea transmission dynamics in the population. At each time step the population in the ODE sub-model was reconciled with the population in the PDE model, with each single model run being initiated by first running the gonorrhoea ODE model to equilibrium before introducing HIV into the population.

Post-sexual debut: HIV

$$\frac{\partial X_{s,g}}{\partial a}+\frac{\partial X_{s,g}}{\partial t}=d\omega_{s,g}X_{0,g}\left(a,t\right)-\left[\lambda_{s,g}\left(a,t\right)+\mu_{g}\left(a\right)\right]X_{s,g}\left(a,t\right)$$

$$\frac{\partial Y_{1 s,g}}{\partial a}+\frac{\partial Y_{1 s,g}}{\partial t}=\lambda X_{s,g}\left(a,t\right)-\left[\sigma_{1}+\mu_{g}\left(a\right)\right]Y_{1 s,g}\left(a,t\right)$$

$$\frac{\partial Y_{2 s,g}}{\partial a}+\frac{\partial Y_{2 s,g}}{\partial t}=\sigma_{1}Y_{1 s,g}-\left[\sigma_{2}+\mu_{g}\left(a\right)\right]Y_{2 s,g}$$

$$\frac{\partial A_{s,g}}{\partial a}+\frac{\partial A_{s,g}}{\partial t}=\left(1-p_{R}\right)\sigma_{3}Y_{3 s,g}\left(a,t\right)+\varphi R_{s,g}\left(a,t\right)-\left[\alpha+\mu_{g}\left(a\right)\right]A_{s,g}$$

$$\frac{\partial R_{s,g}}{\partial a}+\frac{\partial R_{s,g}}{\partial t}=\frac{p_{R}}{2}\sigma_{2}Y_{2 s,g}\left(a,t\right)+p_{R}\sigma_{3}Y_{3 s,g}\left(a,t\right)-\left[\varphi+\mu_{g}\left(a\right)\right]R_{s,g}\left(a,t\right)$$

$$N_{s,g}\left(a,t\right)=X_{s,g}\left(a,t\right)+Y_{1s,g}\left(a,t\right)+Y_{2s,g}\left(a,t\right)+Y_{3s,g}\left(a,t\right)+A_{s,g}\left(a,t\right)+R_{s,g}\left(a,t\right)$$

where:

*X_s,g_(a,t)* = number of people of gender *g* in sexual activity group *s* at age *a*, time *t* who are susceptible to HIV infection;

*Y_1s,g_(a,t)* = number of people of gender *g* in sexual activity group *s* at age *a*, time *t* with primary HIV infection;

*Y_2s,g_(a,t)* = number of people of gender *g* in sexual activity group *s* at age *a*, time *t* with latent HIV infection;

*Y_3s,g_(a,t)* = number of people of gender *g* in sexual activity group *s* at age *a*, time *t* with increased HIV infectivity (pre-AIDS);

*A_s,g_(a,t)* = number of people of gender *g* in sexual activity group *s* at age *a*, time *t* with AIDS;

*R_s,g_(a,t)* = number of people of gender *g* in sexual activity group *s* at age *a*, time *t* who are receiving ARV treatment;

*d* = rate of sexual debut *per annum*;

*μ_g_(a)* = background mortality rate *per annum* experienced by those of gender *g* at age *a*;

*ω_s,g_ =* proportion of the population of gender *g* in sexual activity group *g*;

*λ_s,g_(a,t)* = HIV force of infection experienced by people of gender *g* in sexual activity group *s* at age *a*, time *t*;

*σ_1_* = rate *per annum* of transition from primary to latent HIV infection;

*σ_2_* = rate *per annum* of transition from latent HIV infection to pre-AIDS;

*σ_3_* = rate *per annum* of transition from pre-AIDS toAIDS;

*p_R_=* proportion of people who enter ARV treatment on transition from current infection stage;

*φ* = rate of failure of ARV treatment;

*α* = mortality *per annum* for those suffering from AIDS;

Post-sexual debut: gonorrhoea

$$X'_{s,g}\left(t\right)=D_{s,g}\left(t\right)+\theta Y_{s,g}\left(t\right)+\theta'Y'_{s,g}\left(t\right)-\left[\mu+\lambda'_{s,g}\left(t\right)\right]X'_{s,g}\left(t\right)$$

$$Y_{s,g}\left(t\right)=\left[1-p_{T s,g}\left(t\right)\right]\lambda'_{s,g}\left(t\right)X'_{s,g}\left(t\right)-\left[\mu+\theta\right]Y_{s,g}\left(t\right)$$

$$Y'_{s,g}\left(t\right)=p_{T s,g}\left(t\right)\lambda'_{s,g}\left(t\right)X'_{s,g}\left(t\right)-\left[\mu+\theta'\right]Y'_{s,g}\left(t\right)$$

$$N'_{s,g}\left(t\right)=X'_{s,g}\left(t\right)+Y_{s,g}\left(t\right)+Y'_{s,g}\left(t\right)=\int_{a_{0}}^{a_{1}} N_{s,g}\left(a,t\right) da$$

$$\mu N'_{s,g}\left(t\right)=\int_{a_{0}}^{a_{1}} \mu\left(a\right)N_{s,g}\left(a,t\right) da$$

$$D_{s,g}\left(t\right)=d\omega_{s,g}\int_{a_{0}}^{a_{1}} X_{0,g}\left(a,t\right) da$$

where:

*X΄_s,g_(t)* = number of people of gender *g* in sexual activity group *s* at time *t* who are susceptible to gonorrhoea infection;

*Y_s,g_(t)* = number of people of gender *g* in sexual activity group *s* at time *t* with untreated gonorrhoea infection;

*Y΄_s,g_(t)* = number of people of gender *g* in sexual activity group *s* at time *t* with treated gonorrhoea infection;

*θ* = rate of recovery from untreated gonorrhoea infection;

*θ΄* = rate of recovery from treated gonorrhoea infection;

*λ΄_s,g_(t)* = gonorrhoea force of infection experienced by people of gender *g* in sexual activity group *s* at time *t*;

*p_T s,g_(t)=* proportion of people of gender *g* in sexual activity group *s* at time *t* who are treated on being infected with gonorrhoea;

*a_1_,a_2_* = ages at start and cessation of sexual activity;

Pre sexual debut

$$\frac{\partial X_{0,g}}{\partial a}+\frac{\partial X_{0,g}}{\partial t}=-\left[d+\mu_{g}\left(a\right)\right]X_{0,g}\left(a,t\right)$$

$$X_{0,g}\left(0,t\right)=p_{g}\sum_{s} \int_{a_{3}}^{a_{4}} \nu\left(a\right)X_{s,f}\left(a,t\right) da$$

where:

*X_0,g_(a,t)* = number of people prior to sexual debut of gender *g* in sexual activity group *s* at age *a*, time *t*;

*X_s,f_(a,t)* = number of women post-sexual debut in sexual activity group *s* at age *a*, time *t*;

*ν(a)* = fertility rate *per annum* at age *a*;

*a_3_,a_4_* = ages at start and cessation of fertility;

*p_g_* = proportion of births of gender *g*;

Force of infection - HIV

$$\lambda_{s,m}\left(a,t\right)=w_{Gc}\left[c_{s,m}\left(a,t\right)\Lambda_{s,m}\left(a,t\right)+\hat{c}_{s}\left(a,t\right)\hat{\Lambda}_{s}\left(a,t\right)\right]$$

$$\lambda_{s,g}\left(a,t\right)=w_{Gc}c_{s,g}\left(a,t\right)\Lambda_{s,g}\left(a,t\right)$$

$$w_{Gc}=\frac{X'_{s,g}\left(t\right)+f_{Gc}\left[Y_{s,g}\left(t\right)+Y'_{s,g}\left(t\right)\right]}{N'_{s,g}\left(t\right)}$$

where

$\Lambda_{s,g}\left(a,t\right)$ = component of force of infection for gender *g* (*m* = male), sexual activity group *s*, arising from non-commercial sexual contacts;

$\hat{\Lambda}_{s}\left(a,t\right)$ = component of force of infection for males of sexual activity group *s* arising from commercial sexual contacts with FSW;

*w_Ng_* = weighting representing the effect on the FOI of the proportion of the population with gonorrhoea infection;

$c_{s,g}\left(a,t\right)$ = numbers of new sexual contacts *per annum* by people of gender *g* in sexual activity group *s* at age *a*, time *t*

$\hat{c}_{s}\left(a,t\right)$ = numbers of new sexual contacts with FSW *per annum* by males in sexual activity group *s* at age *a*, time *t*

*f_Ng_* = Factor representing increased infectivity of HIV from contacts with gonorrhoea infection;

and similarly for $\hat{\Lambda}_{s}\left(a,t\right)$ where

*ρ_g,s,s΄_(a,a΄,t)* = contact or mixing matrix representing the proportion of partners at time *t* which people of age *a,* gender *g* and sexual activity level *s* have with people of age *a΄* and activity group *s΄*;

$p_{g,s,s'}^{cdm}\left(t\right)$ = proportion of partnerships by those of gender *g*, activity group *s*, with those in activity group *s΄* in which a condom is used;

*Φ_HIV_, Φ_Ng_* = efficacy of condoms against, respectively, transmission of HIV and gonorrhoea;

*β_1 g,g΄_, β_2 g,g΄_, β_3 g,g΄_* = risk of transmission of HIV in a partnership between a person of gender *g* and an infected person of gender *g΄* in, respectively, the stage of acute infection, of latent or ARV treated infection, and pre-AIDS, where

$$\beta_{i,g,s,s'}\left(a\right)=1-\left(1-B_{i,g}\right)^{\eta_{g,s,s'}\left(a\right)}$$

$$\beta'_{i,g,s,s'}=1-\left(1-B'_{g}\right)^{\eta'_{g,s,s'}}$$

where

*η_g,s,s΄_(a)* = acts per partnership by someone of gender *g*, sexual activity group *s*, at age *a* with someone of sexual activity group *s΄*;

*η΄_g,s,s_* = acts per partnership by someone of gender *g*, sexual activity group *s*, with someone of sexual activity group *s΄*;

*B_i,g_* = risk of HIV transmission per act from an individual of gender *g* in infection stage *i*;

*B΄_g_* = risk of gonorrhoea transmission per act from an individual of gender *g*.

**Contact or mixing matrix**

For contacts between males and non-FSW females the contact matrix is:

$$\rho_{g,s,s'}\left(a,a',t\right)=\varepsilon\delta_{g,s,s',a,a'}+\left(1-\varepsilon\right)P_{g,s,s',a'}\left(t\right)$$

whereas for contacts between FSW and their clients the contact matrix is:

$$\rho_{g,s,s'}\left(a,a',t\right)=P_{g,s,s',a'}\left(t\right)$$

where

*ε* is a weighting factor determining the degree of assortative mixing within age groups

*δ_g,s,s΄,a,a΄_* : corresponds to a fully assortative mixing matrix and

P *_g,s,s΄,a,a΄_(t)* corresponds to a random mixing matrix:

Assortative mixing matrix

for females (*f*)

$$\delta_{f,low,s',A,A'}=\frac{\Pi_{m,s'}}{\sum_{\hat{s}=1}^{\hat{s}=4} \Pi_{m,\hat{s}}}, A=A', s'\in\left\{1,\ldots,4\right\}$$

$$\delta_{f,med,s',A,A'}=\frac{\Pi_{m,s'}}{\sum_{\hat{s}=2}^{\hat{s}=4} \Pi_{m,\hat{s}}}, A=A', s'\in\left\{2,\ldots,4\right\}$$

$$\delta_{f,s,s',A,A'}=rand, s\in\left\{3,\ldots,6\right\}, s'\in\left\{3,4\right\}$$

$$\delta_{f,s,s',A,A'}=0, A\neq A', s\in\left\{1,2\right\}, s'\in\left\{1,\ldots,4\right\}$$

for males

$$\delta_{m,low,s',A,A'}=1, A=A', s'=low$$

$$\delta_{m,med,s',A,A'}=\frac{\Pi_{f,s'}}{\sum_{\hat{s}=1}^{\hat{s}=2} \Pi_{f,\hat{s}}}, A=A', s'\in\left\{1,\ldots,2\right\}$$

$$\delta_{m,s,s',A,A'}=rand, s\in\left\{3,4\right\},s'\in\left\{3,\ldots,6\right\}$$

$$\delta_{m,s,s',A,A'}=0, A\neq A', s\in\left\{1,\ldots,2\right\}$$

where *Π_m,s_* and *Π_f,s_* correspond, respectively, to the number of partnerships (pairings) ‘offered’ by males and females in sexual activity group *s* (1 = low risk males or females, 2 = medium risk males or females, 3 = short-term FSW clients or Benin FSW, 4 = long-term FSW clients or Ghana FSW, 5 = Togo FSW, 6=Nigeria FSW)

*A, A΄* are 5 year age bands (15-19, 20-24, 25-29, 30-34, 35-39, 40-44, 45-49, 50-54, 55-59)

Random mixing matrix

$$\begin{matrix} P_{m,low,s',A'}\left(t\right)=\frac{c_{f,s',A'}\left(t\right)N_{f,s'}\left(a,t\right)}{\int_{A'}^{A'+5} c_{f,s',A'}\left(t\right)N_{f,s'}\left(\alpha,t\right)d\alpha}, s'=low, \\ P_{m,low,s',A'}\left(t\right)=0, s'\neq low, \\ A'\in\left\{15,20,25,30,35,40,45,50,55\right\} \end{matrix}$$

$$P_{m,s,s',A'}\left(t\right)=\frac{c_{f,s',A'}\left(t\right)N_{f,s'}\left(a,t\right)}{\sum_{s'=low}^{s'=med} \int_{A'}^{A'+5} c_{f,s',A'}\left(t\right)N_{f,s'}\left(\alpha,t\right)d\alpha}, s\neq low,s'\in\left\{low,med\right\}$$

$$P_{m,s,s',A'}\left(t\right)=\frac{c_{f,s',A'}\left(t\right)N_{f,s'}\left(a,t\right)}{\sum_{s'=FSW_{1}}^{s'=FSW_{4}} \int_{A'}^{A'+5} c_{f,s',A'}\left(t\right)N_{f,s'}\left(\alpha,t\right)d\alpha}, s\notin\left\{low,med\right\},s'\in\left\{FSW_{1},\ldots,FSW_{4}\right\}$$

$$P_{f,low,s',A'}\left(t\right)=\frac{c_{m,s',A'}\left(t\right)N_{m,s'}\left(a,t\right)}{\sum_{\hat{s}'} \int_{A'}^{A'+5} c_{m,\hat{s'},A'}\left(t\right)N_{m,\hat{s'}}\left(\alpha,t\right)d\alpha}$$

$$P_{f,med,s',A'}\left(t\right)=\frac{c_{m,s',A'}\left(t\right)N_{m,s'}\left(a,t\right)}{\sum_{\hat{s}'} \int_{A'}^{A'+5} c_{m,\hat{s'},A'}\left(t\right)N_{m,\hat{s'}}\left(\alpha,t\right)d\alpha}, s',\hat{s}'\neq low$$

$$P_{f,FSW,s',A'}\left(t\right)=\frac{c_{m,s',A'}\left(t\right)N_{m,s'}\left(a,t\right)}{\sum_{\hat{s}'} \int_{A'}^{A'+5} c_{m,\hat{s'},A'}\left(t\right)N_{m,\hat{s'}}\left(\alpha,t\right)d\alpha}, s',\hat{s}'\notin\left\{low,med\right\}$$

Balancing partner numbers

To ensure partner numbers balance despite relative changes in numbers in each subgroup

$$\begin{matrix} c_{g,s,A}\left(t\right)= \\ c_{g,s,A}\left(0\right)\frac{\sum_{s'} \int_{a_{0}}^{a_{\max}} \left(1-\varphi\right)c_{m,s',A}\left(0\right)N_{m,s'}\left(a,t\right)+\sum_{s''} \int_{a_{0}}^{a_{\max}} \varphi c_{f,s'',A}\left(0\right)N_{f,s''}\left(a,t\right)da}{\sum_{s'} \int_{a_{0}}^{a_{\max}} c_{g,s',A}\left(0\right)N_{g,s'}\left(a,t\right)}, \\ \forall s,s' s''\in\left\{low,med\right\} \end{matrix}$$

$$\begin{matrix} c_{m,s,A}\left(t\right)= \\ c_{m,s,A}\left(0\right)\frac{\sum_{s'} \int_{a_{0}}^{a_{\max}} \left(1-\varphi'\right)c_{m,s',A}\left(0\right)N_{m,s'}\left(a,t\right)da+\sum_{s''} \int_{a_{0}}^{a_{\max}} \varphi'c_{f,s'',A}\left(0\right)N_{f,s''}\left(a,t\right)da}{\sum_{s'} \int_{a_{0}}^{a_{\max}} c_{m,s',A}\left(0\right)N_{g,s'}\left(a,t\right)da} \\ s,s',s''\notin\left\{low,med\right\}, \end{matrix}$$

Force of infection – gonorrhoea

$$\lambda'_{s,m}\left(t\right)=\kappa_{s,m}\left(t\right)\Lambda'_{s,m}\left(t\right)+\kappa'_{s}\left(t\right)\hat{\Lambda'}_{s}\left(t\right)$$

$$\lambda'_{s,g}\left(t\right)=\kappa_{s,g}\left(t\right)\Lambda'_{s,g}\left(t\right)$$

$$\kappa_{s}\left(t\right)=mean \left(c_{s,g}\left(a,t\right),t\right)+\frac{\mathrm{var} \left(c_{s,g}\left(a,t\right),t\right)}{mean \left(c_{s,g}\left(a,t\right),t\right)}$$

$$\kappa'_{s}\left(t\right)=mean\left(\hat{c}_{s}\left(a,t\right),t\right)+\frac{\mathrm{var}\left(\hat{c}_{s}\left(a,t\right),t\right)}{mean\left(\hat{c}_{s}\left(a,t\right),t\right)}$$

where

$\Lambda'_{s,g}\left(t\right)$ = component of force of infection at time *t* for gender *g* (*m* = male), sexual activity group *s*, arising from non-commercial sexual contacts;

$\hat{\Lambda}'_{s}\left(t\right)$ = component of force of infection at time *t* for males of sexual activity group *s* arising from commercial sexual contacts with FSW;

where the contact matrix for gonorrhoea, the proportion of partners at time *t* which people of gender *g*, sexual activity group *s*, have with people of sexual activity group *s΄*, is:

$$\rho'_{g,s,s'}\left(t\right)=\frac{\int_{a_{0}}^{a_{\max}} \left\{\int_{a'_{0}}^{a'_{\max}} \rho_{g,s,s'}\left(a,a',t\right)da'\right\}da}{\int_{a_{0}}^{a_{\max}} \rho_{g,s,s'}\left(a,a',t\right)da}$$

MIGRATION OF FSW

$$\begin{matrix} y_{s}\left(t\right)=\theta_{s}t+K_{s}, t<t_{q_{s}} \\ y_{s}\left(t\right)=\theta_{s}t_{q_{s}}+K_{s}, t\geq t_{q_{s}} \end{matrix}$$

where *t = t_qs_* represents a time point from which HIV prevalence in incoming FSW becomes constant

To take into account entry and exit to the FSW populations of each non-Beninoise nationality in Cotonou, each year outgoing and incoming numbers of FSW of each nationality are subtracted and added as follows:

$$\begin{matrix} Outgoing=\frac{Z_{s,f}\left(a,t\right)}{N_{s,f}\left(a,t\right)}\frac{\Theta_{s,A}^{out}}{\Delta A}\int_{a_{0}}^{a_{\max}} \Omega_{s}^{out}\left(t\right)N_{s,f}\left(\alpha,t\right)d\alpha, A\in\left\{15,20,\ldots,55\right\}, \Delta A=5 \\ Z_{s,f}\left(a,t+1\right)=Z_{s,f}\left(a,t\right)+Outgoing \end{matrix}$$

where

*Z_s,f_(a,t)* corresponds to FSW of nationality (risk group) *s* of age *a* at time *t* for FSW in infection status *X_s,f_(a,t), Y_1s,f_(a,t), Y_2s,f_(a,t), Y_3s,f_(a,t), A_s,f_(a,t), R_s,f_(a,t)* as the case may be; if *Outgoing > Z_s,f_(a,t)* the magnitude of the shortfall for each FSW nationality, $Z_{s,f}\left(a,t\right)-Outgoing=\Xi_{s}\left(a,t\right)$*,* is added to the incoming FSW (i.e. removed) according to the same distribution of infection status as other incoming FSW;

$\Omega_{s}^{out}\left(t\right)$ = proportion of FSW in Cotonou of nationality *s* annually leaving the city at time *t*;

$\Theta_{s,A}^{out}/\Delta A$ = age distribution by 5-year age groups of FSW of nationality *s* leaving Cotonou;

$$Ingoing=\Gamma_{s,A,i}\left(t\right)\frac{\Theta_{s,A}^{in}}{\Delta A}\int_{a_{0}}^{a_{\max}} \Omega_{s}^{in}\left(t\right)N_{s,f}\left(\alpha,t\right)+\Xi_{s}\left(\alpha,t\right)d\alpha$$

where

$\Omega_{s}^{in}\left(t\right)$ = proportion of existing FSW in Cotonou of nationality *s* corresponding to annual influx at time *t* of new FSW of that nationality;

$\Theta_{s,A}^{in}/\Delta A$ = age distribution by 5-year age groups of incoming FSW of nationality *s*;

$\Gamma_{s,A,i}\left(t\right)$ = distribution of infection stages *i* of HIV infection in FSW of nationality *s* and age group A

START & CESSATION OF BENINOIS FSW

Baseline numbers of Benin women starting SW were augmented by replacement of background and AIDS mortality:

$$In_{1}=\int_{a_{0}}^{a_{\max}} \left(\Omega_{s}^{in}\left(t\right)+\mu_{f}\left(a\right)\right)N_{s,f}\left(a,t\right)+\alpha A_{s,f}\left(a,t\right)da$$

where

$\Omega_{s}^{in}\left(t\right)$ = proportion of Beninois FSW in Cotonou corresponding to annual influx at time *t* of Beninois women to FSW;

Baseline numbers of Benin women ceasing SW were:

$$Out_{1}=\int_{a_{0}}^{a_{\max}} \Omega_{s}^{out}\left(t\right)N_{s,f}\left(a,t\right)da$$

where

$\Omega_{s}^{out}\left(t\right)$ = proportion of Beninois FSW annually ceasing FSW at time *t*;

To ensure model numbers of Benin FSW as a proportion of all FSW reflected data on the proportion of FSW from Benin, a correction, *Q*, was added to the baseline values *In_1_* and *Out_1_* according to the difference between model numbers and data for FSW at time *t*:

$$Q=p_{B}\left(t\right)\sum_{s=3}^{s=6} \int_{a_{0}}^{a_{\max}} N_{s,f}\left(\alpha,t\right)d\alpha-\left[\int_{a_{0}}^{a_{\max}} N_{s=3,f}\left(\alpha,t\right)d\alpha+In_{1}-Out_{1}\right]$$

$$In_{2}=In_{1}+Q/2, Out_{2}=Out_{1}-Q/2$$

$$Outgoing=\frac{Z_{s,f}\left(a,t\right)}{N_{s,f}\left(a,t\right)}\frac{\Theta_{s,A}^{out}}{\Delta A}Out_{2}, A\in\left\{15,20,\ldots,55\right\}, \Delta A=5$$

$$Z_{s,f}\left(a,t+1\right)=Z_{s,f}\left(a,t\right)-Outgoing, s=3$$

$$\begin{matrix} Z_{low,f}\left(a,t+1\right)=Z_{low,f}\left(a,t\right)+p_{L}Outgoing \\ Z_{med,f}\left(a,t+1\right)=Z_{med,f}\left(a,t\right)+\left(1-p_{L}\right)Outgoing \end{matrix}$$

$$Ingoing=\frac{Z_{s,g}\left(a,t\right)}{\sum_{s'=low}^{s'=med} X_{s',g}\left(a,t\right)+Y_{1s',g}\left(a,t\right)+Y_{2s',g}\left(a,t\right)+Y_{3s',g}\left(a,t\right)+A_{s',g}\left(a,t\right)+R_{s',g}\left(a,t\right)}\frac{\Theta_{s,A}^{in}}{\Delta A}In_{2}$$

where

*p_B_* = proportion of FSW of Benin origin at time *t*;

*p_L_* = proportion of new FSW drawn from low risk women;

AGE-DISTRIBUTED PARAMETERS

Pseudo-triangular age-distributions of incoming $\Theta_{s,A}^{in}$ and outgoing $\Theta_{s,A}^{out}$ FSW for each nationality (excl Benin??) by 5-year age groups were specified by LHC sampled parameters corresponding to the mode, Μ, and a baseline value, *θ*:

$$\begin{matrix} \Theta'_{s,A}^{out}=\theta+\frac{\left(A-A_{\min-1}\right)}{\left(M-A_{\min-1}\right)}, A\leq M \\ \Theta'_{s,A}^{out}=\theta+\frac{\left(A_{\max+1}-A\right)}{\left(A_{\max+1}-M\right)}, A>M \end{matrix}$$

$$\Theta_{s,A}^{out}=\sum_{A=\min}^{A=\max} \Theta'_{s,A}^{out}$$

and similarly for $\Theta_{s,A}^{in}$.

Other age-distributed rates, $\hat{\Theta}_{s,A}$, with LHC sampled mode, Μ, and baseline value, *θ*, plus an additional parameter, *H*, were calculated as:

$$\begin{matrix} \hat{\Theta}_{s,A}=\theta'+\frac{\left(A-A_{\min-1}\right)H}{\left(M-A_{\min-1}\right)}, A\leq M \\ \hat{\Theta}_{s,A}=\theta'+\frac{\left(A_{\max+1}-A\right)H}{\left(A_{\max+1}-M\right)}, A>M \end{matrix}$$

The relevant rates were: i) male and ii) female sexual debut; rates for low risk males of iii) becoming and iv) ceasing to be FSW clients; for moderate risk males of v) becoming and vi) ceasing to be FSW clients; vii) of low risk males becoming moderate risk; viii) of moderate risk males becoming low risk; of ix) low risk and x) moderate risk becoming FSW; of FSW becoming xi) low risk and xii) moderate risk; xiii) of moderate risk females becoming low risk and xiv) low risk females becoming moderate risk.
