# Supplementary material for: Positive Impact of Increases in Condom Use among Female Sex Workers and Clients in a Medium HIV Prevalence Epidemic: Modelling Results from Project SIDA1/2/3 in Cotonou, Benin
Source: PLoS One. 2014 Jul 21;9(7):e102643. doi: 10.1371/journal.pone.0102643 (PMC4105482; doi:10.1371/journal.pone.0102643)
Supplement: Text S2 — Further modelling details. (DOC) [file pone.0102643.s017.doc]

**Text S2: Further modelling details**

**Demography**

Demographic data relating to Cotonou is limited, and it is not always clear whether they refer to fixed census enumeration districts or whether the boundaries to which the data relate have been expanding over recent years as the city itself has expanded to encompass an area which could be described as ‘Greater Cotonou’ and which forms the large part of the administrative area of Littoral Departement. It has also been necessary to rely on population projections rather than actual census data. It is, therefore, similarly somewhat unclear to what extent comparisons are valid between data and model outputs representing the population of Cotonou (an issue which is largely distinct from the separate question of to what extent the model population is a valid representation of the population of Cotonou). Nevertheless putting this issue to one side, comparisons of model outputs with a number of demographic indicators were made.

The table below compares model outputs with estimated data for: a) the male:female ratio at two time points, and for children and adults aged 0 to 60 years and separately for adults aged 15 to 60 years; b) the ratios of the sizes of the entire male population in 2012 and 2002 up to age 60 and also aged 15 to 60 years, and similarly for females; and c) population growth rates for the four 5 year time periods from 1990 to 2010. Model results for the years 2002 and 2012 were less variable than for the data and the model male to female ratios for all ages lay within the interval bounded by the data for the two time points; for ages 15-60 the model results somewhat underestimated the proportion of males, particularly for 2012, perhaps a result of the data relating to Littoral Departement rather than Cotonou proper, and also perhaps the likely inclusion in the data of short-term migrant male workers associated with rapid urbanisation (Bilsborrow 1992) [1]. The comparison of ratios of population sizes, 2012 and 2002, suggested the model was underestimating somewhat the changes in male population size, particularly for adults and somewhat overestimating changes in female population size, this contrast perhaps again an indication of an effect resulting from short-term migration of male workers [1]. UN data [2] for overall population growth for Cotonou showed a decrease in the rate of population growth followed by an increase over the periods 1990 through to 2010, whereas population growth in the model was much less variable and generally higher than shown in the UN projections.

**Table Example from a single model run of comparison between model outputs and demographic data**

| **Male:female ratio** | |  | **Year** | **Data[3],[4]** | **Model** |
| --- | --- | --- | --- | --- | --- |
| (Age 0-60) | | | 2002 | 0.954 | 0.973 |
|  | |  | 2012 | 0.994 | 0.971 |
| (Age 15-60) | | | 2002 | 0.996 | 0.961 |
|  | |  | 2012 | 1.068 | 0.957 |
| **Ratio population size (2012 vs 2002) of males and females** | | | | | |
|  |  | | **Age range** | **Data[3],[4]** | **Model** |
|  | Male | | 0-60 | 1.438 | 1.420 |
|  |  | | 15-60 | 1.489 | 1.413 |
|  | Female | | 0-60 | 1.380 | 1.422 |
|  |  | | 15-60 | 1.390 | 1.418 |
| **Growth rate** | | |  | **Data[1]** | **Model** |
| (assumes constant growth) | | | |  |  |
|  | 1990-1995 | |  | 2.73 | 3.40 |
|  | 1995-2000 | |  | 2.13 | 3.36 |
|  | 2000-2005 | |  | 2.28 | 3.36 |
|  | 2005-2010 | |  | 3.19 | 3.57 |

While data and model of the age distribution of males and females match quite closely at older ages, a degree of mismatch at younger working ages suggest a process of inward migration to the city not incorporated in the model, and may be suggestive of inward migration of young families [5].

In summary while the simplification within the model of the complex demographic processes operating in a city such as Cotonou inevitably give rise to model results which do not fully reflect heterogeneities in observe demography, it is considered that the model does capture the demography sufficiently well to provide a satisfactory platform upon which to base the investigation of the transmission dynamics of HIV in Cotonou. In saying this the authors also bear in mind that heterogeneities in the demography of the general population are less significant in a setting where the HIV epidemic is concentrated in FSW and their clients as in Cotonou.

**Age of sexual debut**

Children in the model were sexually inactive prior to age 15, and joined the sexually active population at age- and gender-specific rates which were constant over time (Fig S4). Thus the inactive proportion of the population declined with age. In a similar way movements of the population between risk groups (including to and from FSW) also occurred at age- and gender-specific rates distributed by age (Fig S4); both these and rates of debut were sampled at the fitting stage to reflect the uncertainty in parameter assumptions due to data limitation.

**FSW**

The model incorporated transnational migration of FSW into and out of Cotonou, although in many instances appropriate data were lacking. The modelling attempted to overcome the lack of data by using LHS both to sample directly plausible ranges for the parameters concerned and, indirectly, the age-distributions of these processes by sampling ranges for parameters determining shapes of the triangular distributions employed in the model to simulate peaked age distributions of migration.

SIDA1/2/3 data show the proportions of the different FSW nationalities in Cotonou at different time points (Table 1). However it is not known to what extent changes in FSW migration in the intervening periods were relatively smooth or erratic (e.g. due to economic perturbations), and compared with the aggregated FSW prevalence, the disparities between model fits to the data for individual FSW nationalities may well be a reflection of the fact that inward and outward migrations of foreign FSW are likely to have been strongly influenced by short- or long-term changes in economic circumstances both in Benin and in the source countries. It seems highly plausible that such economic drivers might result in ‘spikes’ at different times in rates of inward and outward migrations so that, without much more detailed migration data (or perhaps inclusion of an algorithm relating relevant economic trends to migration) in what is already a complex model, it will be challenging to arrive at closer fits using the phenomenological approach used here. Moreover, anecdotal evidence suggests that some FSW of Benin origin, particularly in the earlier years of the intervention, claimed to be of foreign nationality most probably for reasons of stigma, so that a proportion of Ghanaian, Nigerian and Togolese FSW would in fact have been Beninese. Such concealment of national origin could provide one explanation for why model fits to the data for individual nationalities in some instances were less satisfactory compared to the fits for the aggregated FSW prevalence.

Notwithstanding the apparent success of the modelling in representing the influence of FSW migration patterns on HIV prevalence in Cotonou, the importance of the contribution of time-dependent patterns of transnational migration by FSW towards changes in HIV prevalence and incidence remains to be fully evaluated. The fact that local circumstances in the countries or regions of origin are likely to be the predominating factors driving inward FSW migration, implies the need for much additional data in order to be able to reliably capture the influence of the ebb and flow of FSW migrations on HIV prevalence.

As noted above, unlike the demographic fluctuations observed in closed populations, the volume of short-term migrations associated with FSW is likely to fluctuate quite widely over relatively short time scales according to socio-economic circumstances in the source country as well as in Cotonou itself and the rest of Benin. For example there has been anecdotal evidence to suggest that Nigerian schoolgirls may make repeated short term visits to Benin for SW to obtain money for school fees. Appropriate data collected ad hoc might allow the capture of essential recent past dynamics of cross-border migrations for FSW, but projecting this into the future for the purpose of investigating epidemic dynamics becomes highly challenging as it would be necessary, for example, to forecast future patterns of socio-economic change in the major source countries as the basis of some algorithm to predict migrations for the purpose of sex work. At a more detailed practical level it would be necessary to know what proportion of migrants had previously practised SW in their home regions, the distribution in these region of HIV prevalence by age in females, FSW and previously non-FSW, in the age groups travelling to Cotonou for SW, the age distribution of migrants arriving for sex work and how this changes over time, the age distributions of time spent in sex work in Cotonou and the proportions making repeat visits to Cotonou for sex work and intervals between visits.

**Modelling N.gonorrhoea treatment**

During SIDA1/2/3 screening for gonorrhoea was carried out during visits to each location 3 times per year from the start of the intervention to 2006 and twice per year for 2007-2008. Bearing in mind that gonorrhoea is an infection of generally relatively short duration, this frequency of visits did not necessarily allow identification and treatment of all episodes of this infection. Thus it was assumed, in addition to the possibility of diagnosis during periodic visits by the intervention team, that during the intervening periods also symptomatic cases would be treated based on the assumption that 25% of gonorrhoea infections in females were symptomatic. The additional strong assumption was made that the proportion of FSW seeking treatment for symptoms would be the same as females in the general population.

**Targets used in model calibration**

The lower end of the target ranges was defined as the lower bound of the 99% confidence interval (LCB) minus the difference between a specified fraction (0.75) of the LCB and the mean, and similarly for the upper range.

**References**

1. Bilsborrow RE (1992) Population growth, internal migration, and environmental degradation in rural areas of developing countries. European Journal of Population 8:125-148

2. United Nations Population Division (2009) World Urbanization Prospects: The 2009 Revision Population Database. United Nations: Available: <http://esa.un.org/wup2009/unup/index.asp?panel=2>. Accessed 27 August 2013

3. Institut National de la Statistique et de l’analyse Economique (2012) La Population du Benin 2000-2004. Available: [http://www.insae-bj.org/2012/doc/Notre%20Annuaire/2000_2004/LA_POPULATION_DU_BENIN_2000_2004.pdf](http://www.insae-bj.org/2012/doc/Notre Annuaire/2000_2004/LA_POPULATION_DU_BENIN_2000_2004.pdf) . Accessed: 27 August 2013.

4. Institut National de la Statistique et de l’analyse Economique (2012) Projection de la Population du Littoral, Projections Départementales. Available: <http://www.insae-bj.org/2012/doc/Publications/Projection_Population_Benin_par_Departement_2002_A_2030.pdf>. Accessed: 27 August 2013.

5. Rogers A, Castro LJ, Lea M (2005) Model Migration Schedules: Three Alternative Linear Parameter Estimation Methods. Mathematical Population Studies 12:17–38.
